# Effects of Dietary Astragalus Polysaccharide Supplementation on the Th17/Treg Balance and the Gut Microbiota of Broiler Chickens Challenged With Necrotic Enteritis

**DOI:** 10.3389/fimmu.2022.781934

**Published:** 2022-02-21

**Authors:** Bochen Song, Peng Li, Shaojia Yan, Yan Liu, Mingkun Gao, Huiyuan Lv, Zengpeng Lv, Yuming Guo

**Affiliations:** ^1^ State Key Laboratory of Animal Nutrition, College of Animal Science and Technology, China Agricultural University, Beijing, China; ^2^ Department of Animal Science, Shandong Agricultural University, Taian, China; ^3^ Centre Research Institute, Beijing Centre Biology Co., Ltd., Beijing, China

**Keywords:** necrotic enteritis, astragalus polysaccharide (APS), Th17/Treg, gut microbiota, gut metabolome, broiler chickens

## Abstract

This study aimed to investigate the effects of dietary astragalus polysaccharide (APS) supplementation on the immune function, gut microbiota and metabolism of broiler chickens challenged with necrotic enteritis (NE). Two hundred forty Arbor Acres broiler chicks (one day old) were randomly assigned using a 2 × 2 factorial arrangement into two groups fed different levels of dietary APS (0 or 200 ppm of diet) and two disease challenge groups (control or NE challenged). The results showed that NE infection significantly increased FCR, mortality rate, Th17/Treg (Th17 cells% in blood and ileum, Th17/Treg, IL-17 and IL-17/IL-10 in blood), NO, lysozyme activity and IL-1β in blood, intestinal immune cell proportion and activity (Tc%, Treg% and monocyte phagocytic activity in ileum), intestinal inflammatory cytokines (TLR2, NF-κB, TNF-α and IL- 6) gene expression levels, and the number of *Clostridium perfringens* in cecum. NE infection significantly reduced body weight gain, thymus index, lymphocyte proliferation activity in blood and ileum, villus height and V/C in jejunum, Th cells% and Mucin2 gene expression in ileum. Dietary APS supplementation significantly increased body weight, feed intake, proportion of immune cells (T cells in blood and Tc, Treg in ileum), lymphocyte proliferation activity, V/C in jejunum, and ZO-1 gene expression in ileum. Dietary APS supplementation significantly reduced FCR and mortality rate, Th17/Treg, Th17%, intestinal pathology scores, intestinal inflammatory cytokine gene expression levels, and the number of *Clostridium perfringens* in cecum. In addition, broilers challenged with NE significantly increased *Staphylococcus* and *Turicibacte*r and reduced α diversity of microbiota in ileum. Dietary APS supplementation significantly increased α diversity, *Romboutsia*, *Halomonas*, propionic acid, butyric acid, formononetin, taurine, cholic acid and equol and downregulated uric acid, L-arginine and serotonin in ileum. Spearman’s correlation analysis revealed that *Romboutsia, Turicibacter, Staphylocpccus, Halomonas, Streptococcus, Escherichia-Shigella, Prevotella*, uric acid, L-arginine, jerivne, sodium cholate and cholic acid were related to inflammation and Th17/Treg balance. In conclusion, APS alleviated intestinal inflammation in broilers challenged with NE probably by regulating intestinal immune, Th17/Treg balance, as well as intestinal microbiota and metabolites.

## Introduction

Necrotic enteritis (NE) is an enterotoxic disease caused by type A or type C *Clostridium perfringens*. The mortality rate of necrotizing enteritis is high, and the mortality rate can be as high as 50% when an acute disease occurs, especially when it occurs in chicks, while chronic necrotizing enteritis will significantly reduce the growth performance of chickens, causing intestinal ulcers, erosions, and other adverse effects, seriously affecting the health of poultry and causing serious economic losses to the poultry farming industry (1, 2).

Helper T cells 17 (Th17) and regulatory T cells (Treg) are two closely related T cell types. They play key roles in promoting the immune response and suppressing immunity, respectively. Th17 cells and Tregs coordinate and balance to maintain the normal immune function of animals. Previous studies have found that IL-1β is significantly increased when intestinal inflammation occurs in mice and mediates chronic intestinal inflammation by promoting the accumulation of IL-17A-secreting innate lymphoid cells and Th17 cells (3). Studies have also reported that mice challenged with enterotoxigenic *Escherichia coli* significantly increase the proportions of Th17 and Treg cells in the spleen and intestinal mucosal lymph nodes, upregulate the mRNA and protein expression of RORα in the intestinal mucosal lymph nodes, and downregulate the mRNA and protein expression of Foxp3 (4). In addition, a study found that TNBS- and DSS-induced colitis in mice can significantly increase the level of IL-17 in the intestine (5). Chickens challenged with *coccidia* significantly increase the proportions of Th17 cells in the spleen and caecal tonsils (6). Th17/Treg cells have been widely studied in mammals with ulcerative colitis, but there are no reports about changes in the Th17 cell ratio in broilers challenged with necrotic enteritis.

Chickens with healthy intestines can not only improve the digestion and absorption rate of feed nutrients, but also resist the invasion of intestinal pathogenic bacteria and reduce economic losses caused by disease deaths and complications, which are the key factors to ensure good growth performance of chickens (7). Factors that affect gut health include diet, pathogens, environment, and management. Plant polysaccharides can “escape” the absorption of the small intestine, reach the second half of the intestine and be fermented by the microbiota, and exhibit anti-inflammatory, immune regulation, intestinal barrier protection, antioxidant functions and other activities through a microbiota-dependent or independent mechanism, and thus has become a research hotspot (8–10).

Astragalus polysaccharide (APS) can regulate the immune function of the body. With immune-enhancing and anti-inflammatory properties, it has a unique two-way regulatory ability. Studies have found that the addition of 4 mg/kg APS to the diet has an immunomodulatory effect on broilers challenged by LPS and can reverse the negative effects of LPS. This includes decreases in daily weight gain and feed intake and decreases in the number of intraepithelial lymphocytes and villus height in the jejunum. APS can also reduce the feed conversion rate, serum NO levels and the heterophilic cell/lymphocyte (H: L) ratio (11). Studies in mice have found that APS may effectively alleviate TNBS-induced experimental colitis in rats by restoring the number of Treg cells and inhibiting the level of IL-17 in Pyle’s nodes (12). However, the effect of APS on Th17/Treg balance has not been reported in poultry.

According to reports, gut microbiota and metabolites can regulate the ratio of Th17 and Treg cells and the levels of cytokines in animal intestines (13). The molecular weight of APS will be large, and it is difficult for the endogenous enzymes produced in the animal intestine to completely degrade it into monosaccharides; thus, APS can easily reach the back of the intestine, where it is utilized by microorganisms. Therefore, in addition to its similar structure to LPS, APS can directly regulate the immune function of the host and may also indirectly affect the immune function of the host by regulating the gut microbiota and metabolites. Previous studies have found that adding 200 ppm APS to hen diets increases the relative abundance of caecal *Firmicutes* and *Lactobacteriaceae* (14). The addition of γ-irradiated Astragalus polysaccharide (IAPS) to the diet of immunosuppressive broilers significantly increased the caecal microbial α diversity. After adding IAPS to broiler diets, there were lower proportions of *Faecalibacterium*, *Bacteroides*, and *Butyricicoccus* and higher proportions of *Ruminococcaceae UCG-014*, *Negativibacillus*, *Shuttleworthia*, *Sellimonas* and *Lamina RF39_norank* (15). The study found that adding 0.5% crude *Astragalus* to the hen’s diet significantly increased *Lactobacillus* in faeces and significantly reduced the relative abundance of *Romboutsia* sp (16).. However, the effects of APS on the ratios of Th17 cells and Tregs in broilers with necrotizing enteritis and the key gut microbes and metabolites that it regulates have not been extensively studied. In view of the ability of APS to regulate the T cell immune response, the importance of T cell immunity in enteritis, and the ability of gut microbiota to regulate T cell subsets, in this study, we explored whether dietary APS supplementation might regulate CD4+ T cell immunity and the gut microbiota and metabolism of broilers with necrotic enteritis. We hypothesize that dietary APS supplementation will regulate the TLRs-NFκB signalling pathway and gut microbiota and metabolism, leading to a dominant position of Tregs, regulating the balance of Th17/Treg, thereby reducing intestinal inflammation and preventing tissue damage.

## Materials and Methods

### Astragalus Polysaccharide

The APS used in this study was purchased from Beijing Centre Biology Co., Ltd. (Beijing). The APS was extracted from the *Astragalus membranaceus* (Fisch.) Bge. var. *mongholicus* (Bge.) Hsiao. The purity of APS is 80%. The molecular weight of APS was determined by LC-MS, ranging from 8 to 160,000 Daltons. According to the best dosage recommended by Beijing Centre Biology Co., Ltd., the added dosage of APS in this study was 200ppm.

### Experimental Animals and Diets

Two hundred forty Arbor Acres broiler chicks (one day old) were randomly assigned using a 2 × 2 factorial arrangement into two groups fed different levels of dietary APS (0 or 200 ppm of diet) and two disease challenge groups (control or NE challenged). According to the recommendations of the National Research Council (NRC 2012), drug-free corn-soybean meal diets should be formulated to meet or exceed the nutritional requirements of broilers (**Table 1**). A double-layer, three-dimensional chicken coop was used, and standard management procedures were followed throughout the experiment. The chickens had free access to clean water and feed.

**Table 1:** Ingredients and composition (calculated and analyzed nutrients) of the experimental diets^a^ (%, unless otherwise noted, as-fed basis).

Item	d 1 to 21	d 22 to 31
Composition, %		
Corn (7.8% CP)	51.38	60.02
Soybean meal (46% CP)	40.71	25.54
Corn protein flour	0.00	5.66
Soybean oil	3.75	3.32
Wheat flour	0.00	2.00
CaHPO_3_.2H_2_O	1.86	1.33
Stone powder (37%)	1.24	1.14
Sodium chloride	0.35	0.35
DL-Methionine (98%)	0.20	0.070
L-Lysine HCL (98%)	0.00	0.19
Vitamin premix^b^	0.03	0.03
Mineral premix^c^	0.20	0.20
Choline chloride (50%)	0.25	0.16
Sandoquin (Ethoxyquinoline)	0.030	0.00
Calculated nutrient levels^d^		
Metabolizable energy (Kcal/kg)	2928.97	3100.00
Crude protein	21.76	20.00
Calcium	1.01	0.90
Available phosphorus	0.44	0.35
Lysine	1.14	1.00
Methionine	0.54	0.40

a Diets were in mash form.

b Vitamin premix provided per kg of complete diet: vitamin A(retinylacetate), 9,500 IU; vitamin D3 (cholecalciferol), 2,500 IU; vitamin E(DL-a-tocopherol acetate), 30 IU; vitamin K3(menadione sodium bisulfate), 2.65 mg; vitamin B12(cyanocobalamin), 0.025 mg; biotin, 0.30 mg; folic acid, 1.25 mg; nicotinic acid, 50 mg; d-pantothenic acid, 12 mg; pyridoxine hydrochloride, 6.0 mg; riboflavin, 6.5 mg; thiamine mononitrate, 3.0 mg.

c Mineral premix provided per kg of complete diet: iron, 80 mg; copper, 8 mg; manganese, 100 mg; zinc, 80 mg; iodine, 0.35 mg; selenium, 0.15 mg.

d Calculated value based on the analysis of experimental diets.

### Establishment of the Necrotic Enteritis Model

A broiler subclinical NE model was constructed based on previous research experience (17). The method was as follows: feed was cut 8 hours in advance, and then on the 13th day of age, broiler chickens in the challenge group were orally inoculated with 30 times the dose of attenuated coccidia IV vaccine, 33,000 sporangia per chicken (containing Eimeria acervulina PAHY strain, Eimeria giant PMHY strain, Eimeria tenella PTMZ strain, and Eimeria tenella PNHZ strain Attenuated oocysts, Foshan Biotechnology Co., Ltd., Foshan, China). In the control group, 1 mL of sterile PBS was administered orally at the same time. Feed was cut 8 hours in advance, and then at the age of 17 to 23 for seven consecutive days of challenge, all the broiler chickens in the challenge group were orally inoculated with 1 mL of freshly prepared *Clostridium perfringens* CVCC2030 at a concentration of 1×10^9^ CFU/mL (China Veterinary Drug Control Center, Beijing, China). Broilers in the control group were force-fed the same volume (1 mL) of sterilized thioglycolate fluid medium (FT, CM801, Beijing Luqiao Technology Co., Ltd.) at the same time point.

### Sample Collection and Index Determination

#### Growth Performance

At 13, 25, and 31 days of age, feed and broilers were weighed in cages to calculate the average daily feed intake (ADFI), average daily gain (ADG), feed conversion rate (FCR) and mortality rate. All chicken coops were checked every day for broiler deaths.

#### Immune Organ Index

At 2 days and 8 days after infection, namely, 25 and 31 days of age, 6 healthy chickens were randomly selected for each treatment (1 for each replicate). After weighing, the chickens were euthanized by intravenous injection of 50 mg/kg body weight sodium pentobarbital solution under the wings. The thymus, spleen and bursa of fabricius were collected and weighed. Immune organ index (g/kg) = immune organ weight (g)/body weight (kg).

### Peripheral Blood Mononuclear Cell Isolation

The isolation of peripheral blood mononuclear cells (PBMC) was conducted as previously described (18) using density gradient centrifugation with Ficoll-Paque Plus following the manufacturer’s guidelines. Briefly, six healthy chickens (1 bird per replicate) were randomly selected from each treatment group on d 25 and 31. Heparinized blood samples were collected from the wing vein and then diluted 1:1 with sterile calcium- and magnesium-free Hank’s balanced salt solution (CMF-HBSS, Sigma). The diluted samples were placed on ice and then carefully layered into a tube containing an equal volume of Ficoll lymphocyte separation medium (Histopague-1077, Tianjin HaoYang Biological Manufacture Co., Ltd., China) to form a distinct layer above the Ficoll. Following centrifugation at 400 ×*g* for 30 min at room temperature, the white flocculent material on the interface between the plasma and the lymphocyte separation medium was transferred to a clean tube using a sterile transfer pipette. The lymphocyte suspension was washed 3 times with RPMI 1640 (Invitrogen Corp., Grand Island, NY, USA) incomplete culture medium and then resuspended in 2 mL of RPMI 1640 complete culture medium supplemented with 5% (vol/vol) fetal calf serum, 0.5% penicillin (final concentration, 100 U/mL), 0.5% streptomycin (final concentration, 100 mg/mL), and 1% N-(2-hydroxyethyl)-piperazine-N-2-ethanesulfonic acid (HEPES, final concentration, 24 mmol/L; Amresco 0511, Amresco Inc., Cleveland, OH). The live cells were detected using the Trypan blue dye exclusion technique and a microscope (DM6000B, Leica Microsystems, Wetzla, Germany). The cell suspensions were diluted to a final concentration of 1 × 10^7^ cells/mL in RPMI 1640 medium for subsequent analysis.

### Isolation of Ileum Propria Lymphocytes (LPLs)

Separation solution was prepared by adding 5% FBS (Gibco, United States), 1 mmol/L DTT (Biosharp, South Korea) and 10 mmol/L HEPES (Biosharp, South Korea) to D-Hank’s solution without calcium magnesium phenol red (HyClone, United States). Digestion solution was prepared by adding 5% FBS (Gibco, USA), 0.25% pancreatin (15090046, Gibco, USA), 0.1% collagenase I (17100, Gibco, USA) and 100 U/mL DNAse I (Thermo Scientific, USA) and incubating at 37°C for 5 min.

For cleaning, 1 g of the anterior ileum (1 cm after the yolk antrum) was cut out. All samples were the same weight. The intestinal tube was cut longitudinally along the mesentery side. The small intestine was turned so that the mucosa faced outward, after which it was rinsed gently in Hank’s until the chyme was completely rinsed, and it was then cut into approximately 0.5 cm intestinal tissue fragments laterally. For separation, 5 ml of separation solution was added to a 50-ml centrifuge tube, the tube was placed in a constant temperature shaking box and shaken at 37°C (250 r/min) for 15 minutes and vortexed for 30 seconds, and the intestinal tissue fragments were then filtered through a 200-mesh cell sieve, after which the intestinal tissue fragments were collected in a 50-ml centrifuge tube. To chop the intestinal tissue fragments, they were moved to a petri dish and ophthalmic scissors were used to cut the tissue 100 times into a muddy shape. Five millilitres of digestion solution was then added to a 50-ml centrifuge tube, and the samples were placed in a constant temperature shaking box and shaken (250 r/min) at 37°C for 30–45 minutes. The samples were then vortexed for 30 seconds, the intestinal tissue fragments were filtered through a 300-mesh (48 μm) cell sieve, and the filtrate was collected in a sterile 7 ml centrifuge tube and centrifuges at 4°C (400 x g or 3000 r/min) 10 min (1500–2000 r/min is too low, making it easy to lose many cells, as the enzyme is soluble in water, and there are many washing steps in the differential centrifugation method). The supernatant was discarded, and the pellet was resuspended in 2 ml of RPMI 1640 (or D-Hank’s, wash). Samples were then centrifuged (400 x g or 3000 r/min) at 4°C for 10 min, the supernatant was discarded, and the pellet was resuspend in 2 ml of RPMI 1640. The organ tissue lymphocyte separation solution was extracted by differential centrifugation (2500 r/min), washed repeatedly and resuspended, and finally resuspended and cultured with complete RPMI 1640. According to the method of the organ lymphocyte extraction kit, Ficoll-Paque Plus (Histopague-1077, Tianjin HaoYang Biological Manufacture Co., Ltd., China) was used for density gradient centrifugation, and the subsequent method was the same as that of peripheral blood lymphocyte separation.

### Ilea Lymphocytes Are Stimulated to Produce Cytokines and Stain Intracellular Cytokines (Th17)

For the intracellular staining of IL-17A, the volume of the isolated ileal lymphocytes was adjusted to 1 ml, and the concentration was adjusted to 2 x 10^6^ cells/mL. Two microliters of cell activation stimulator (423304, Biolegend, San Diego, California, USA), was added (each 100 μL of cell stimulator contains 2.5 mg/ml Brefeldin A, 40.5 µM PMA, 669.3 µM ionomycin), and the cells were incubated in a CO_2_ incubator at 37°C for 6 hours. The cells were then centrifuged at 350 x g for 5 minutes, the supernatant was discarded, and the activated cells were collected. Then, 2.5 mL of cell staining buffer (420201, Biolegend, San Diego, California, USA) was added to the cell pellet, mixed by vortexing or pipetting, and centrifuged at 350 x g for 5 minutes. The supernatant was then discarded, and the cells were collected by centrifugation again and resuspended in complete RPMI-1640, after which they were ready for analysis to detect the surface markers or intracellular IL-17 expression of the cells.

### Peripheral Blood and Ileum Mononuclear Cell Proliferation

A 3-(4,5-dimethylthiazol)-2,5-diphenyltetrazolium bromide (MTT, Sigma Chemical Co., St. Louis, MO) assay was used to determine the peripheral blood and ileum lymphocyte proliferation response. Briefly, 100 μL of the PBMCs suspension and 100 μL of RPMI 1640 in the absence or presence of 90 μg/mL concanavalin A (Con A; C2613, Sigma Chemical, Co.) or 50 μg/mL lipopolysaccharide (L3129, Sigma Chemical, Co.) were added to a 96-well microtiter plate (Costar 3599, Corning, Inc., Corning, NY). The cultures were set up in triplicate. After a 68-h incubation in a 5% CO_2_ incubator (MCO-18AIC CO_2_ incubator, Sanyo Electric Biomedical Co. Ltd., Tokyo, Japan) at 39°C, MTT was added to each well at a final concentration of 5 mg/mL. The cells were incubated for an additional 4 h, and then, 100 μL of 10% sodium dodecyl sulfate dissolved in 0.04 mol/L HCl solution was added to each well to lyse the cells and solubilize the MTT crystals. Finally, the absorbance value of each sample was determined using an automated ELISA reader (model 550 Microplate Reader, Bio-Rad Pacific Ltd., Hong Kong, China) at 570 nm. The stimulation index (SI) for each sample was calculated based on the following formula:


SI=(Absorbance value of mitogen−Stimulated cells)/(Absorbance value of media without mitogen).

### Phagocytic Activity of Mononuclear Lymphocytes in Peripheral Blood and Ileum

The phagocytic activity levels of peripheral blood and ileal mononuclear lymphocytes were measured by the neutral red assay method. In short, peripheral blood PBMCs were collected, and the number of cells was adjusted to 2×10^6^/ml with RPMI 1640 medium. Then, 100 μL of cell suspension was incubated in a 96-well cell culture plate for 2 hours (with 3 replicate wells), the supernatant was discarded, and 200 μL/well of 0.1% neutral red solution (N299163, Shanghai Aladdin Biochemical Technology Co., Ltd., China) was added, and the cells were further cultured for 2 h. The supernatant was then discarded, and any remaining neutral red was washed away with PBS (3 times). Cell lysate was added at 200 μl/well (ethanol:acetic acid=1:1), kept in the dark at 4°C for 12 h, and the OD value was measured at 550 nm.

### Determination of Lymphocyte Subsets in Peripheral Blood and Ileum PBMCs by Flow Cytometry

As mentioned earlier (19, 20), flow cytometry was used to analyze CD3+, CD4+, CD8+, CD25+, IL-17A+ and monocytes/the percentage of macrophage+ cells. In short, primary antibodies against mouse anti-chicken CD45-FITC (8270-02), mouse anti-chicken CD3-Alexa Fluor^®^ 700 (8200-27), mouse anti-chicken CD4-APC (8210-11), mouse anti-chicken CD8α-Pacific Blue™ (8220-26), mouse anti-chicken monocyte/macrophage-PE (8420-09), mouse anti-chicken CD4-Pacific Blue™ (8210-26) (Southern Biotechnology Associates Inc., Birmingham, A), human anti-chicken CD25-Alexa Fluor^®^ 647 (HCA173A647, Bio–Rad Antibodies) and rat anti-mouse IL-17A-PE (506904, Biolegend, San Diego, California, USA) in PBS (pH 7.2) were used. A volume of 100 μL of PBMCs (2×10^6^ cells) was added to a 1.0-mL Eppendorf tube, and 25 μL of diluted primary monoclonal antibody (1:100 dilution) and negative isotype control IgG (mouse IgG1κ)-SPRD, mouse IgG1κ-FITC and mouse IgG1κ-R-PE) were used for staining. After incubating for 45 minutes at room temperature, the cells were washed twice with cold PBS and centrifuged at 1,800 x g for 30 minutes to remove unbound primary antibody. A total of 300 μL of haemolysin solution diluted in PBS (1:25) was added to each tube. Finally, the cells were washed twice, and the final volume was 500 μL. Five-color flow cytometry analysis was performed using a Coulter XL (Beckman Coulter Corp., Fullerton, CA) at Xiyuan Hospital of Traditional Chinese Medicine, China Academy of Chinese Medical Sciences. Then, the percentages of CD3+ T, CD4+ T, CD8+ T, CD25+ T, IL-17A+ T and monocyte/macrophage cells in PBMCs and LPL were calculated.

### Serum NO, Lysozyme Activity, Cytokine, Immunoglobulin and Mucosal sIgA Contents

A commercial ELISA kit (Genorise Scientific Inc., Paoli, USA) was used to determine the serum levels of IL-1β, IL-4, IL-10, IL-17 and IFN-γ. According to the instructions, a chicken IgG ELISA kit (E30-104, Bethyl Laboratories Inc., Montgomery, TX, USA) was used to determine the level of IgG in the serum. Serum IgG levels were determined using a commercial ELISA kit (IDEXX Laboratories Inc., Westbrook, Maine, USA) according to the protocol recommended by the manufacturer. Serum NO and lysozyme activities were measured using commercial ELISA kits (A013-2-1; A050-1-1, Nanjing Jiancheng Institute of Bioengineering, Nanjing, China). Ileal mucosa sIgA was measured using a commercial ELISA kit (YM-SQ2632, Shanghai Yuan Mu Biotechnology Co., Ltd., Shanghai, China).

### Intestinal Lesion Score

According to a method previously described, the intestinal lesion score was determined: the lesions were observed, and the lesion scores were evaluated (21).

### Intestinal Tissue Morphology

Fixed intestinal tissue was dehydrated and embedded in paraffin. Tissue sections (thickness of 4 μm) were stained with haematoxylin and eosin (HE, Olympus BX50; Tokyo, Japan). Observed by a Leica microscope (Wetzlar, Germany, ModelDMi8), 10 intact intestinal villi were randomly selected for each slice. Image-ProPlus (version 6.0) software was used to measure the height of each intestinal villus and its corresponding crypt depth and to calculate the ratio of the two. The height of the villi is defined as the vertical distance from the tip of the villi to the villi-crypt junction, and the crypt depth is the vertical distance from the villi-crypt junction to the base of the crypt.

### Pathology Score

The fixed jejunum tissue samples were dehydrated and embedded in paraffin. A serial microtome (Leica Microsystems K.K., Tokyo, Japan) was used to slice the embedded tissue into thin slices (4 μm) and mount it on a polylysine-coated glass slide (Boster Corporation, China) following haematoxylin-eosin (HE) staining. Histopathological examination was performed by optical microscopy and a pathological image analysis system (Leica Qwin, Jiangsu, China). The pathology of the jejunum was determined according to the following criteria: (1) inflammation (0~3 points): 0=no inflammatory cell infiltration; 1=mild inflammatory cell infiltration; 2=moderate inflammatory cell infiltration; and 3=severe inflammatory cell infiltration. (2) Damage (0~3 points): 0=no damage; 1=damage to the mucosal layer; 2=damage to the mucosal layer and submucosa; 3=transparent and disappearance of the cell wall. (3) Crypt damage (0~4 points): 0=no damage to crypts; 1 = 1/3 crypts disappeared; 2 = 2/3 crypts disappeared; 3=only the epithelial surface was intact; 4=crypts and all the epithelium on the surface disappeared. The sum of the three scores is the final intestinal pathology score.

### Jejunum and Ileum Gene Expression

At 31 days of age, molecular samples of the jejunum and ileum were quick-frozen in liquid nitrogen and then transferred to a -80°C low-temperature freezer to determine the expression levels of immune function-related genes in the spleen and ileum. TRIzol reagent was used to extract total jejunum RNA, and a NanoDrop ultra-micro-calculation protein analyzer was used to determine the quality and concentration of RNA. The reagent kit used in the reverse transcription step was the PrimeScript™ RT reagent Kit with gDNA Eraser (Perfect Real Time) from Takara. The cDNA obtained after reverse transcription was subjected to real-time fluorescent quantitative PCR on an ABI 7500 real-time fluorescent quantitative PCR instrument with the primers shown in **Table 2**. The fluorescence quantification kit was Takara’s SYBR^®^ Premix Ex Taq™ II (Tli RNaseH Plus), with GAPDH as the internal reference, and the results are expressed as 2 (-△△CT).

**Table 2:** Sequences of the oligonucleotide primers used for quantitative real-time PCR^a^.

Gene^b^	Primer sequence^3^(5′to3′)^c^	Accession NO.
*AhR*	F: CACCTACGCCAGTCGCAAGC	NM_001323184.1
	R: CCTGTGCCTCTTGGATGGATTGG	
*Caspase1*	F: GTGCTGCCGTGGAGACAACATAG	XM_015295935.1
	R: AGGAGACAGTATCAGGCGTGGAAG	
*chCD25*	F: AAGACAAACCCAAAGCCC	NM_204596
	R: CTCAGAGAGGCATGTGGGAC	
*Foxp3*	F: GCACACCTCTCAATGCTGCT	XP_015148603.2
	R: CTAGGTTGCCCAGAGTGGGA	
*GAPDH*	F: AGAACATCATCCCAGCGTCC	NM_204305
	R: CGGCAGGTCAGGTCAACAAC	
*IFN-γ*	F: AAAGCCGCACATCAAACACA	NM_205149.1
	R: GCCATCAGGAAGGTTGTTTTTC	
*IgA*	F: ACCACGGCTCTGACTGTACC	S40610.1
	R: CGATGGTCTCCTTCACATCA	
*IL-1β*	F: TGGGCATCAAGGGCTACA	NM_204524.1
	R: CGGCCCACGTAGTAAATGAT	
*IL-4*	F: GTGCCCACGCTGTGCTTAC	NM_001007079.1
	R: AGGAAACCTCTCCCTGGATGTC	
*IL-6*	F: GATCCGGCAGATGGTGATAA	NM_204628.1
	R: AGGATGAGGTGCATGGTGAT	
*IL-10*	F: CGCTGTCACCGCTTCTTCA	AJ621614
	R: TCCCGTTCTCATCCATCTTCTC	
*IL-17A*	F: CTCCGATCCCTTATTCTCCTC	AJ493595
	R: AAGCGGTTGTGGTCCTCAT	
*IL-17F*	F: TGAAGACTGCCTGAACCA	JQ776598
	R: AGAGACCGATTCCTGATGT	
*IL-22*	F: TGTTGTTGCTGTTTCCCTCTTC	AJ617782.1
	R: CACCCCTGTCCCTTTTGGA	
*iNOS*	F: GAACAGCCAGCTCATCCGATA	U34045
	R: CCCAAGCTCAATGCACAACTT	
*IRAK4*	F: TGGTTCGCTGCTTGACAGACTTG	XM_015281244.2
	R: TGATGCCATTCGCAGTACCTTGAG	
*Lysozyme C*	F: GACGATGTGAGCTGGCAG	NM_205281
	R: GGATGTTGCACAGGTTCC	
*Mucin2*	F: TTCATGATGCCTGCTCTTGTG	XM_421035
	R: CCTGAGCCTTGGTACATTCTTGT	
*NF-kB*	F: TGGAGAAGGCTATGCAGCTT	NM_205134.1
	R: CATCCTGGACAGCAGTGAGA	
*NLRP3*	F: GGTTTACCAGGGGAAATGAGG	NM_001348947.1
	R: TTGTGCTTCCAGATGCCGT	
*Occludin*	F: AGTTCGACACCGACCTGAAG	NM_205128.1
	R: TCCTGGTATTGAGGGCTGTC	
*pIgR*	F: ATTTGTCACCACCACAGCCA	NM_001044644
	R: GAGTAGGCGAGGTCAGCATC	
*RORα*	F: AAATGCCTTGCTGTGGGGATGTC	NM_001289887.1
	R: GATGATCTCGCTGCTGCTGCTG	
*TGF-β1*	F: GCCGACACGCAGTACACCAAG	NM_001318456.1
	R: GCAGGCACGGACCACCATATTG	
*TLR2*	F: ACCTTCTGCACTCTGCCATT	NM_204278.1
	R: TGTGAATGAAGCACCGGTAA	
*TLR4*	F: GATGCATCCCCAGTCCGTG	NM_001030693
	R: CCAGGGTGGTGTTTGGGATT	
*TNF-α*	F: CCCCTACCCTGTCCCACAA	AY765397.1
	R: TGAGTACTGCGGAGGGTTCAT	
*TRIF*	F: TCAGCCATTCTCCGTCCTCTTC	NM_ 496065.1
	R: GGTCAGCAGAAGGATAAGGAAAGC	
*ZO-1*	F: ACAGCTCATCACAGCCTCCT	XM_015278981.1
	R: TGAAGGGCTTACAGGAATGG	

a Primers designed using Primer Express software (Sangon Biotech, Shanghai, China).

b AhR, aryl hydrocarbon receptor; ChCD25, chicken cluster of differentiation 25; Foxp3, forkhead transcription factor p 3; GAPDH, glyceraldehyde-3-phosphate dehydrogenase; IFN-γ, interferon-γ; IgA, immune globulin A; IL-1β, interleukin-1β; iNOS, inducible nitric oxide synthase; IRAK4, interleukin-1 receptor associated kinase-4; Lyz C, lysozyme C; MUC2, Mucin-2; NF-κB, nuclear factor kappa-β; NLRP3, NOD-like receptor family proteins 3; pIgR, polymeric immunoglobulin receptor; RORα, retinoic acid related-orphan receptors α; TGF-β1, transforming growth factor-β1; TLR2, toll-like receptor 2; TNF-α, tumor necrosis factor α; TRIF, TIR-domain-containing adaptor inducing interferon-β; ZO-1, zonula occludens-1.

c F, forward; R, reverse.

### Caecal Microbial Count

Under aseptic conditions, caecum specimens from broiler chickens 2 and 8 days after infection were collected, quickly frozen in liquid nitrogen and stored at -20°C for caecal bacterial count. The specific method was as follows: the caecum was placed on ice (approximately 4°C) to thaw, and 0.3 g was weighed on a balance on a clean bench and placed in a 5-ml sterile centrifuge tube. Then, 2.7 ml of sterile saline was added for a 10-fold dilution, and the sample was shaken and mixed on a micro shaker and allowed to stand for 10 minutes. Then, 0.3 ml of the supernatant was moved to a sterile centrifuge tube, and sterile saline was used to perform gradient dilutions of 10^2^, 10^3^, 10^4^, 10^5^, 10^6^, 10^7^, 10^8^. One hundred microlitres of the solution from each dilution tube was inoculated on the corresponding selective medium, and the plate was spread until the solution was dry on the medium. After culturing under the corresponding conditions, 30–300 colonies were selected for bacterial count. Among them, *Clostridium perfringens* was selected on tryptone-sulfite-cycloserine (TSC) medium (CM138, Beijing Luqiao Technology Co., Ltd., Beijing, China) and cultured under anaerobic conditions at 37°C. Plates were counted after 24 hours. Lactic acid bacteria were counted on MRS agar medium (CM188, Beijing Luqiao Technology Co., Ltd., Beijing, China), and the culture conditions were 5% CO_2_ and 37°C for 24 hours. The logarithm of the number of bacteria per gram of caecal content (log10 CFU/g) was used to express the results.

### DNA Extraction and High-Throughput Sequencing

Bacterial DNA was extracted from ileal digesta with a QIAamp DNA Stool Mini Kit (Qiagen Inc., V alencia, CA) according to the manufacturer’s protocol. The concentrations of DNA extracts were measured on a NanoDrop 2000 spectrophotometer (Thermo Scientific, MA, USA). The V4 region of the bacterial 16S rRNA gene was amplified with the barcoded primer pair 515F/806R (515F: 5′-GTG CCA GCM GCC GCG GTA A-3′, 806R: 5′-GGA CTA CHV GGG TWT CTA AT-3′) according to previously described methods (22). After amplification, PCR products run on a 2% agarose gel and were purified using a QIAquick Gel Extraction Kit (Qiagen, Germany). Pyrosequencing of 16S rDNA was performed on an Illumina HiSeq2500 PE250 platform (Illumina, San Diego, USA) at Novogene Bioinformatics Technology Co. Ltd. (Beijing, China).

### Sequence Processing and Bioinformatics Analysis

Raw tags were generated by merging paired-end reads using FLASH software (v1.2.7) (23). Highquality clean tags were obtained by QIIME (v1.7.0) analysis (24), and chimera sequences were removed to obtain effective tags by using the UCHIME algorithm (25). Sequences were analyzed by UPARSE software (v7.0.1001) and clustered into operational taxonomic units (OTUs) at a similarity level of 97% (26). Each OTU was annotated with the Greengenes database (27). Rarefaction curve and Venn diagram were created using R software (v2.15.3). Analysis of microbial alpha diversity was conducted using QIIME software (24) with Python scripts. Beta diversity was evaluated by principal component analysis (PCA) to show the differences of bacterial community structures, and the significance of separation was tested *via* ANOSIM using R (v2.15.3). PICRUSt analysis was used to predict the functional potential of bacteria communities (28). OTUs were normalized by copy number, and metagenome prediction was further categorized into Kyoto Encyclopedia of Genes and Genomes (KEGG) at levels 2 and 3 (29).

### Short-Chain Fatty Acids of Ileal Contents

Ileal chyme (0.5 g) was weighed in a 10-mL plastic centrifuge tube, 8 ml of deionized water was added, and the samples were ultrasonically shaken for 30 minutes and centrifuged at 8000 rpm at 4°C for 10 minutes. The supernatant was diluted ten times and filtered with a 0.22-μm filter. Twenty-five microlitres of the filtrate was collected, and an ICS-3000 high-performance ion chromatograph (Dionex, USA) was used to detect SCFAs and lactic acid by conductivity. Organic acids were separated under gradient conditions in an AS11 analytical column (250 mm×4 mm) and a AG11 guard column: the gradient used potassium hydroxide as the carrier, 0–5 min, 0.8–1.5 mM; 5–10 min, 1.5–2.5 mM, 10–15 min, 2.5 mM, flow rate 1.0 mL/min.

### Detection of Metabolite Composition of Ileal Contents by Non-Targeted Metabolome

The sample detection and data analysis were all completed by Tianjin Nuohe Zhiyuan Technology Co., Ltd. The metabolite extraction process was as follows: (1) To 100 μL of ileum content, 350 μL of extract (methanol: acetonitrile: water volume ratio=2:2:1, methanol and acetonitrile were both chromatographic grade, purchased from Merck) was added, and then 20 μL of internal standard L-2-chlorophenylalanine (CAS#: 103616–89–3, ≥98%; purchased from Shanghai Hengbai Biotechnology Co., Ltd.) was added. The samples were vortexed and mixed for 30 seconds and then sonicated under ice-water bath conditions for 10 min. (2) The samples were incubated at -20°C for 1 h and then centrifuged at 13,000 rpm for 15 min at 4°C. (3) After carefully removing 400μL of supernatant to a 1.5 mL centrifuge tube, the extract was then dried in a vacuum concentrator. (4) Then, 100 μL of extract (equal volume of acetonitrile and water) was added to the dried extract for reconstitution, and the samples were vortexed for 30 s and ultrasonicated for 10 min in an ice water bath. (5) The samples were then centrifuged at 12,000 rpm at 4°C for15 min. (6) Finally, 60 μL of supernatant was carefully removed and placed in a 2-mL injection bottle, and 10 μL of each sample was mixed into a quality control sample, while 60 μL was taken for computer testing.

The system was used to analyze the metabolome components of the ileum content using an Agilent 1290 ultrahigh-performance liquid chromatograph in a series ABSciexTripleTOF6600 high-resolution mass spectrometer. The column used was an ACQUITYUPLCBEHAmide column (1.7 μm2.1*100 mm) purchased from Waters. The mobile phase conditions were 25 mM ammonium acetate and 25 mM ammonia solution (A) and 100% acetonitrile (B). The gradient items for the analysis of the ileal contents sample were: 0–0.5 min, 5%A, 95%B; 0.5–7 min, 5–35%A, 95–65%B; 7–8 min, 35–60%A, 65–40%B; 8–9 min, 60%A, 40%B; 9–9.1 min, 60–5% A, 40–95%B; 9.1–12 min, 5%A, 95%B; the flow rate was 0.5 mL/min, and the injection volume was 2 μL. The mass spectrometry conditions were as follows: AB6600TripleTOF mass spectrometer, which can perform primary and secondary mass spectrometry data based on the IDA function under the control of the software (AnalystTF1.7, ABSciex). In each data acquisition cycle, the strongest signals with greater than 100 molecules are screened out. The ions were collected corresponding to the secondary mass spectrum data. Bombardment energy: 35 eV, 15 secondary spectra every 50 ms. The ESI ion source parameters were set as follows: atomization pressure (GS1): 60 Pa, auxiliary pressure: 60 Pa, air curtain pressure: 30 Pa, temperature: 550°C, and spray voltage: 5500 V (positive ion mode) or -4500 V (negative ion mode). QC samples with RSD<30% were screened, and a feature yield rate>80% was required to ensure good system stability.

The data were first converted to mzXML format using MSconventer, and XCMS (XCMS1.41.0) was used for peak search, peak alignment and other data processing. Then, the data processing and matching of substance identification were performed, and the xcms4dda and xcms4lipid developed by Nuovo Zhiyuan based on XCMS were used. The program and self-built library were processed, minfrac was set to 0.5, and the cut-off was set to 0.8. First, the secondary data were screened. The screening principle is that as long as one forward and one reverse signal is identified, the peak is retained. Second, the peaks of the first- and second-level data are matched, that is, the peaks of the first-level data corresponding to the second-level data are found, and matching is performed according to mztolerance ± 25 ppm. Data analysis included three parts: basic data analysis and personalized data analysis. The goal of basic data analysis is to perform univariate analysis (UVA) and multivariate analysis (MVA) on the qualitative and quantitative results of the metabolome to screen for metabolites with significant differences. Univariate statistical analysis includes data preprocessing (first simulating the missing values in the original data; the numerical simulation method is the minimum one-half method to fill, and then using the total ion current of each sample for normalization), Student’s t-test and analysis of variance. Multivariate statistical analysis includes principal component analysis (PCA) and partial least squares regression analysis (Partial Least Squares Discrimination Analysis, PLS-DA), with differential compound screening and identification. Personalized data analysis is a series of bioinformatics analyses on metabolites with significant differences on the basis of basic data analysis, including KEGG annotation analysis of different metabolites and metabolic pathway analysis.

### Statistical Analysis

SPSS 20.0 software was used to perform the statistical analysis on each group of data. The GLM process was used for statistical analysis. When the interaction was significant, one-way analysis was used, and Duncan’s multiple comparison analysis was used for differences between treatments. *P* < 0.05 was considered significant, and P values between 0.05 and 0.10 were classified as trends.

The spearman rank correlation coefficient was used for the evaluation of the correlation analysis of the variables and microbes in the broiler chickens.

## Results

### Growth Performance, Mortality Rate and Immune Organ Index

**Figure 1:**
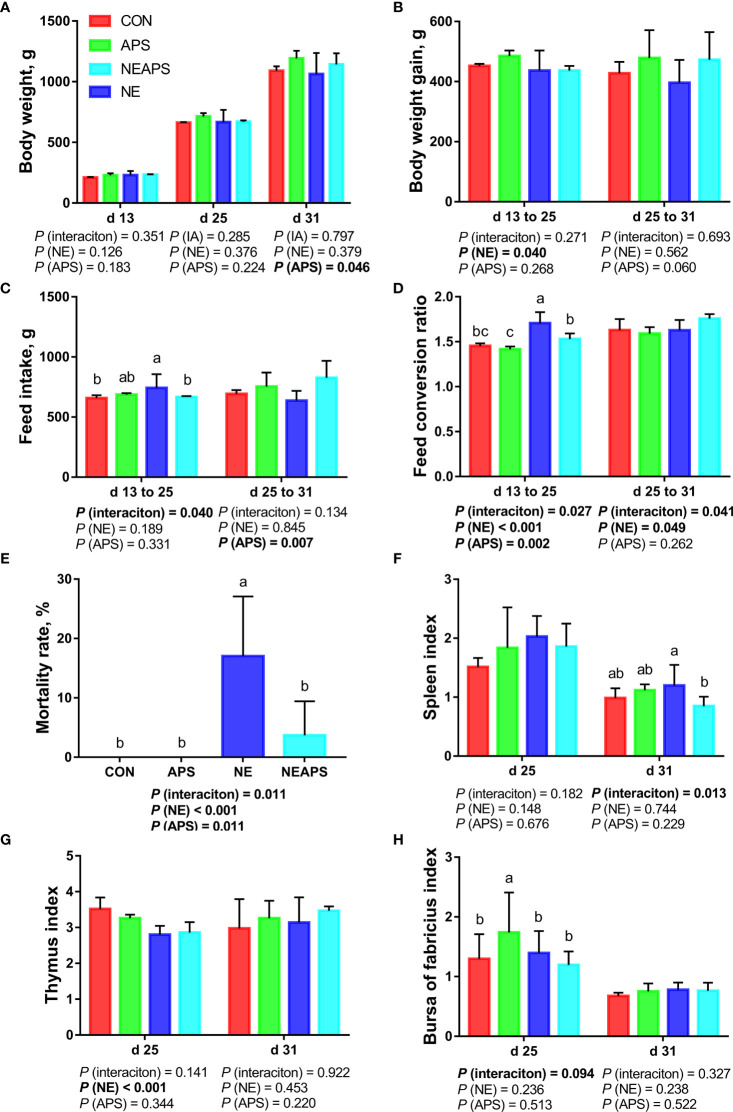
Effects of dietary astragalus polysaccharides supplementation on growth performance, mortality rate and immune organ index of broiler chickens challenged with necrotic enteritis. The body weight **(A)**, body weight gain **(B)**, feed intake **(C)** and feed conversion ratio **(D)** were analyzed by weighted. The mortality rate **(E)** was calculated as the ratio of the number of broilers that died in each cage to the total number of broilers on d 13 to 31. The spleen index **(F)**, thymus index **(G)** and bursa of fabricius index **(H)** were analyzed by weighted. All graphs are presented as mean, with the standard deviation (SD) shown *via* the whiskers. The main effect and interaction effects were analyzed using the general linear model (GLM) procedure, with the *P* values for the main effects written out below each plot. The one-way ANOVA and multiple comparisons were performed when interactive effects differed significantly. The lowercase letters on the bar charts indicate significant differences (*P <*0.05). CON, control group; APS, APS group; NE, NE infection group; NEAPS, APS infected with NE group.

**Figure 1** shows that compared with the unchallenged birds, NE-challenged birds had significantly reduced body weight gain on d 13–25 and mortality on d 13-d 31 (*P* < 0.05) and significantly increased FCR on d 13–25 and d 25–31 (*P* < 0.05). Compared with the control group, dietary APS supplementation alleviated the negative impact of NE on growth performance, significantly reduced the FCR on d 13–25 and the mortality rate on d 13–31 (*P* < 0.05), and significantly increased the average weight on d 31 and the d 25–31 feed intake (*P* < 0.05). There was a tendency to increase body weight gain on d 25–31 (0.05 < *P* < 0.1). Compared with the unchallenged birds, NE-challenged birds had a significantly reduced d 25 thymus index (*P* < 0.05). Compared with the NE group, APS supplementation significantly reduced the spleen index (*P* < 0.05). Compared with the control group, APS supplementation significantly increased the bursa index (*P* < 0.05).

### Peripheral Nonspecific Immune Function and Humoral Immune Function

**Figure 2:**
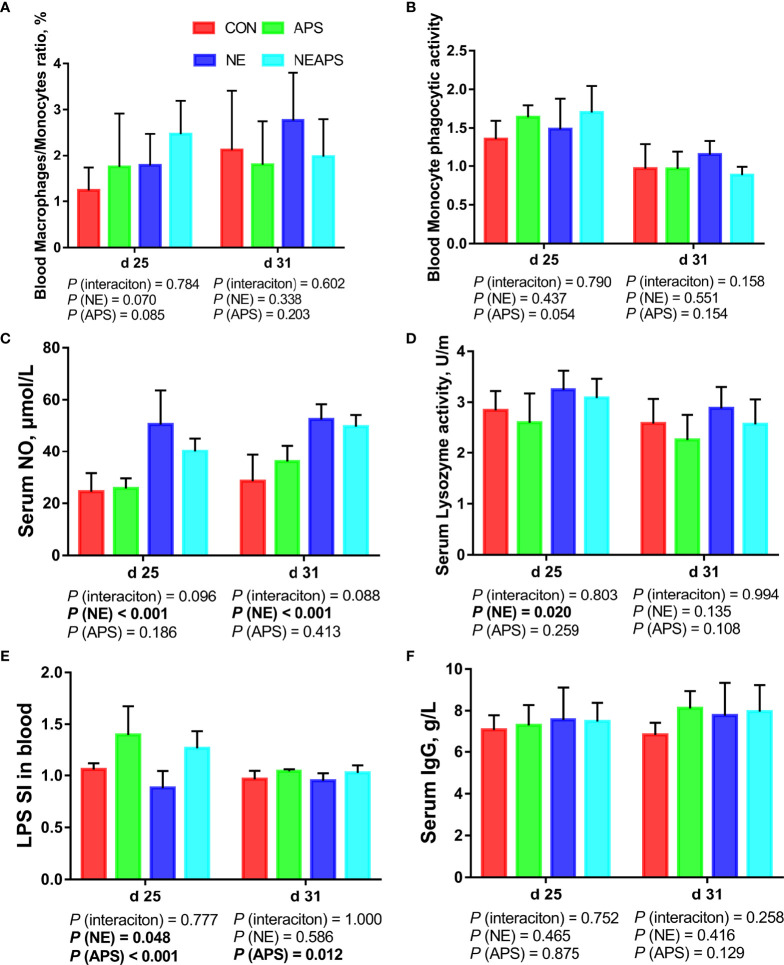
Effects of dietary astragalus polysaccharides supplementation on peripheral nonspecific immune function and humoral immune function of broiler chickens challenged with necrotic enteritis. The frequencies of Mononuclear/Macrophage **(A)** of peripheral blood lymphocytes were analyzed by flow cytometry. The phagocytic activity of Monocytes **(B)** was analyzed by neutral red method. The levels of NO **(C)**, lysozyme activity **(D)**, and IgG **(F)** were analyzed by ELISA kit. Peripheral blood lymphocytes were stimulated with lipopolysaccharide (LPS) **(E)**, and the stimulation index (SI) was calculated as described in the Materials and Methods section. All graphs are presented as mean, with the standard deviation (SD) shown *via* the whiskers. The main effect and interaction effects were analyzed using the general linear model (GLM) procedure, with the *P* values for the main effects written out below each plot. The one-way ANOVA and multiple comparisons were performed when interactive effects differed significantly. CON, control group; APS, APS group; NE, NE infection group; NEAPS, APS infected with NE group.

**Figure 2** shows that compared with the unchallenged birds, NE-challenged birds had significantly increased d 25 and d 31 serum NO levels and d 25 lysozyme activity (*P* < 0.05). There was a tendency to improve the phagocytic function of monocytes (0.05 < *P* < 0.1). Compared with the unchallenged birds, NE-challenged birds had significantly reduced proliferation activity of B lymphocytes in peripheral blood on d 25 (*P* < 0.05), while dietary APS supplementation increased the proliferation activity of B lymphocytes in peripheral blood on d 25 (*P* < 0.05).

### Peripheral Cellular Immune Function

**Figure 3:**
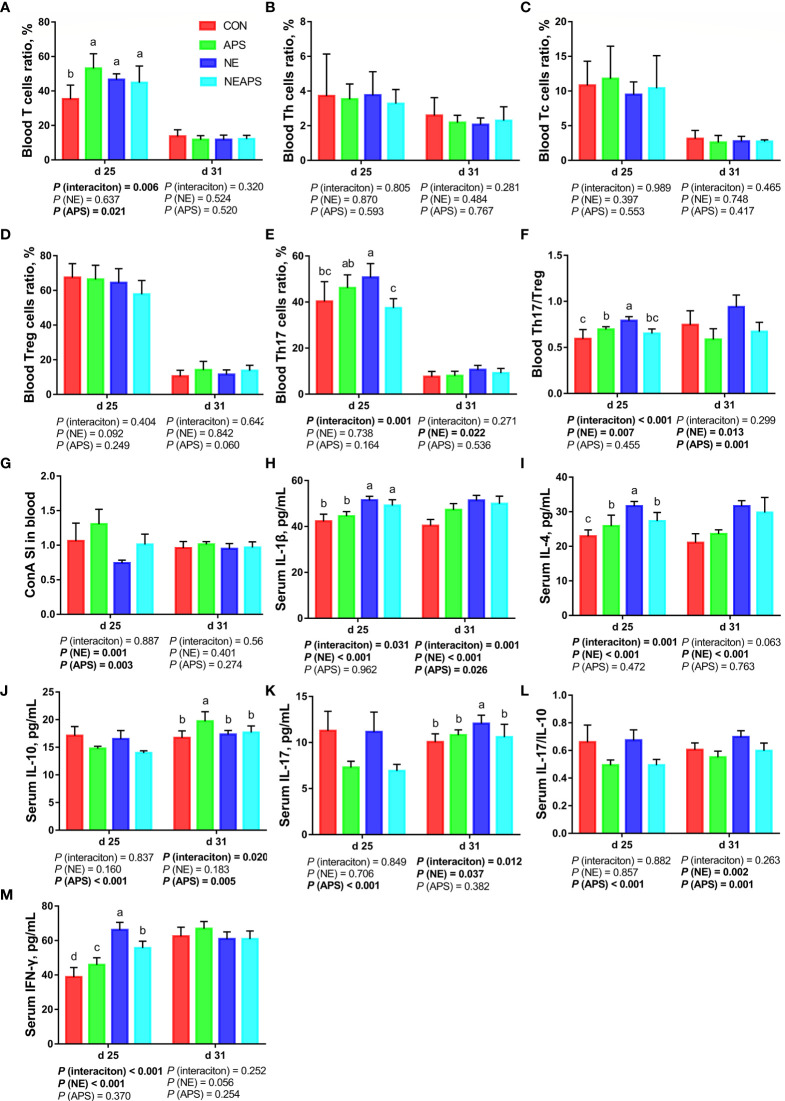
Effects of dietary astragalus polysaccharides supplementation on peripheral cellular immune function of broiler chickens challenged with necrotic enteritis. The frequencies of T **(A)**, Th **(B)**, Tc **(C)**, Treg **(D)** and Th17 **(E)** of peripheral blood lymphocytes were analyzed by flow cytometry. The Th17/Treg **(F)** of peripheral blood lymphocytes was calculated by dividing the proportion of Th17 cells by the proportion of Treg cells. Peripheral blood lymphocytes were stimulated with concanavalin A (ConA) **(G)**, and the stimulation index (SI) was calculated as described in the Materials and Methods section. The levels of IL-1β **(H)**, IL-4 **(I)**, IL-10 **(J)**, IL-17 **(K)**, IL-17/IL-10 **(L)** and IFN-γ **(M)** in serum were analyzed by ELISA kit. All graphs are presented as mean, with the standard deviation (SD) shown *via* the whiskers. The main effect and interaction effects were analyzed using the general linear model (GLM) procedure, with the *P* values for the main effects written out below each plot. The one-way ANOVA and multiple comparisons were performed when interactive effects differed significantly. The lowercase letters on the bar charts indicate significant differences (*P <*0.05). CON, control group; APS, APS group; NE, NE infection group; NEAPS, APS infected with NE group.

As shown in **Figure 3**, compared with the unchallenged birds, NE-challenged birds significantly increased the proportion of Th17 cells in peripheral blood on d 31 and the proportion of Th17/Treg cells on d 25 and d 31 (*P* < 0.05). There was a tendency to increase the number of d 25 peripheral blood mononuclear macrophages and decrease the proportion of Treg cells (0.05 < *P* < 0.1). Compared with the control group, dietary APS supplementation significantly increased the proportion of T cells in peripheral blood on d 25 (*P* < 0.05) and significantly reduced the Th17/Treg cells at 31 days of age (*P* < 0.05). Compared with the unchallenged birds, NE-challenged birds had significantly reduced proliferation activity of T lymphocytes in peripheral blood on d 25 (*P* < 0.05), while dietary APS supplementation increased the proliferation activity of T lymphocytes in peripheral blood on d 25 (*P* < 0.05). Compared with the unchallenged birds, NE-challenged birds had significantly increased d 25 serum cytokine (IL-1β, IL-4 and IFN-γ) and d 31 serum cytokine (IL-1β, IL-4, IL-17 and IL-17/IL-10) levels (*P* < 0.05). Compared with the control group, dietary APS supplementation significantly reduced d 25 serum cytokines (IL-10, IL-17 and IL17/IL-10) and d 31 IL-17/IL-10 levels (*P* < 0.05) and increased d 31 IL-1β and IL-10 levels (*P* < 0.05). There was a tendency to increase the ratios of d 25 peripheral blood mononuclear macrophages and d 31 Treg cells (0.05 < *P* < 0.1).

### Intestinal Lesion Score, Pathology Score and Intestinal Tissue Morphology

**Figure 4:**
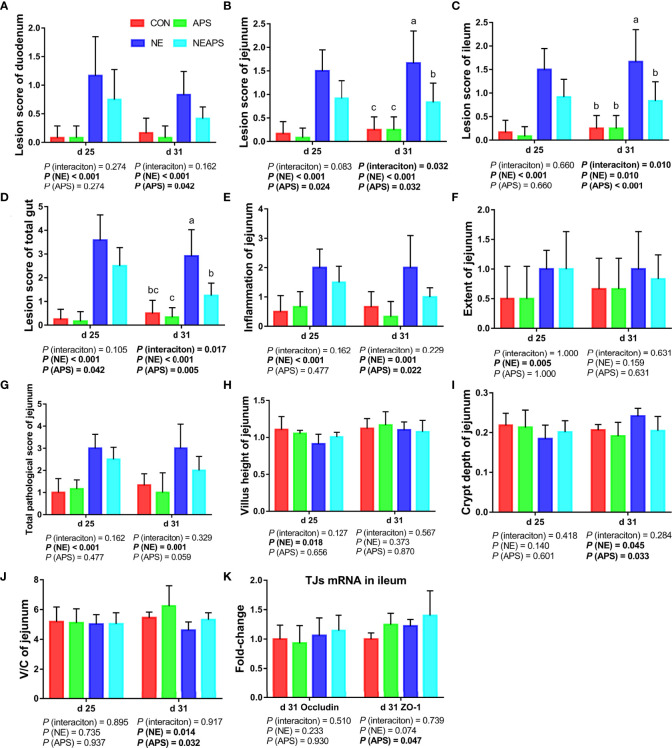
Effects of astragalus polysaccharide supplementation on intestinal lesion score, pathology score and intestinal tissue morphology of broiler chickens challenged with necrotic enteritis. The lesion scores of duodenum **(A)**, jejunum **(B)**, ileum **(C)** and total gut **(D)** were analyzed as described in the Materials and Methods section. The inflammation of jejunum **(E)**, extent of jejunum **(F)** and total pathological score of jejunum **(G)** were analyzed as described in the Materials and Methods section. The villus height of jejunum **(H)** and crypt depth **(I)** of jejunum were analyzed by HE staining. The V/C of jejunum **(J)** was calculated by dividing the villus height by the crypt depth. The mRNA levels of *Occludin* and *ZO-1* in ileum **(K)** were analyzed by RT-PCR. All graphs are presented as mean, with the standard deviation (SD) shown *via* the whiskers. The main effect and interaction effects were analyzed using the general linear model (GLM) procedure, with the *P* values for the main effects written out below each plot. The one-way ANOVA and multiple comparisons were performed when interactive effects differed significantly. The lowercase letters on the bar charts indicate significant differences (*P <*0.05). CON, control group; APS, APS group; NE, NE infection group; NEAPS, APS infected with NE group.

**Figure 4** shows that compared with the unchallenged birds, NE-challenged birds had significantly increased scores of the duodenum, jejunum, the ileum and total score of the intestine of broilers at d 25 and d 31 (*P* < 0.05). Compared with the control group, dietary APS supplementation significantly reduced the scores of d 25 jejunum and d 31 duodenum, jejunum, and ileum and total score of the intestine (*P* < 0.05). Compared with the unchallenged birds, NE-challenged birds significantly improved the inflammatory cell infiltration and pathological score of the jejunum of d 25 and d 31 broilers and the degree of jejunum injury at d 25 (*P* < 0.05). Compared with the control group, dietary APS supplementation significantly reduced the inflammatory cell infiltration in the jejunum of d 31 broilers and had a tendency to decrease the pathological score of the d 31 jejunum (*P* < 0.05). Compared with the unchallenged birds, NE-challenged birds had significantly reduced d 25 jejunum villus height and d 31 V/C (*P* < 0.05) and a significantly increased d 31 jejunal crypt depth (*P* < 0.05). Compared with the control group, dietary APS supplementation significantly reduced the depth of jejunal crypts in broilers at d 31 (*P* < 0.05) and significantly increased the V/C (*P* < 0.05). Compared with the control group, dietary APS supplementation significantly increased the mRNA expression of ZO-1 in ileum on d 31 (*P* < 0.05).

### Intestinal Nonspecific Immune Function and Humoral Immune Function

**Figure 5:**
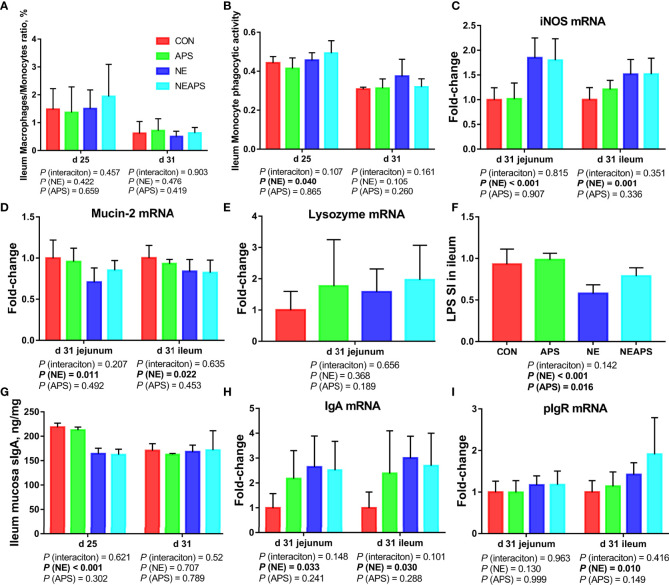
Effects of dietary astragalus polysaccharides supplementation on intestinal nonspecific immune function and humoral immune function of broiler chickens challenged with necrotic enteritis. The frequencies of Mononuclear/Macrophage **(A)** of ileum lymphocytes were analyzed by flow cytometry. The phagocytic activity of Monocytes **(B)** was analyzed by neutral red method. The mRNA levels of *iNOS* **(C)**, *Mucin-2* **(D)**, *lysozyme* **(E)**, *IgA* **(H)** and *pIgR* **(I)** in jejunum and ileum were analyzed by RT-PCR. Ileum lymphocytes were stimulated with lipopolysaccharide (LPS) **(F)**, and the stimulation index (SI) was calculated as described in the Materials and Methods section. The level of sIgA of ileum mucosa **(G)** was analyzed by ELISA kit. All graphs are presented as mean, with the standard deviation (SD) shown *via* the whiskers. The main effect and interaction effects were analyzed using the general linear model (GLM) procedure, with the *P* values for the main effects written out below each plot. The one-way ANOVA and multiple comparisons were performed when interactive effects differed significantly. CON, control group; APS, APS group; NE, NE infection group; NEAPS, APS infected with NE group.

As shown in **Figure 5**, compared with unchallenged birds, NE-challenged birds had significantly improved the phagocytic function of monocytes at d 25. Compared with the unchallenged birds, NE-challenged birds had significantly increased *iNOS*, *IgA* and *pIgR* mRNA levels (*P* < 0.05) and significantly reduced *Mucin-2* mRNA levels in jejunum and ileum at d 31 (*P* < 0.05). Compared with unchallenged birds, NE-challenged birds had significantly reduced proliferation activity of B lymphocytes in the ileum at d 25 (*P* < 0.05) and significantly improved the phagocytic function of monocytes at d 25 (*P* < 0.05). Compared with the control group, dietary APS supplementation improved the proliferation activity of B lymphocytes in the ileum at d 25 (*P* < 0.05). Compared with the unchallenged birds, NE-challenged birds had significantly reduced sIgA levels in the ileal mucosa on d 25 (*P* < 0.05).

### Intestinal Cellular Immune Function

**Figure 6:**
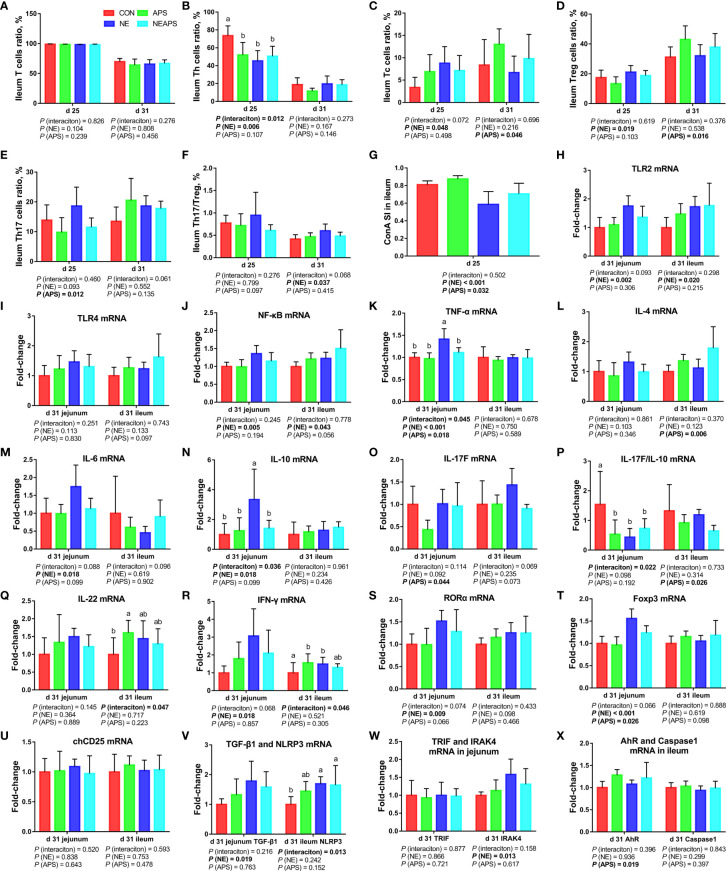
Effects of dietary astragalus polysaccharides supplementation on intestinal cellular immune function of broiler chickens challenged with necrotic enteritis. The frequencies of T **(A)**, Th **(B)**, Tc **(C)**, Treg **(D)** and Th17 **(E)** of ileum lymphocytes were analyzed by flow cytometry. The Th17/Treg **(F)** of ileum lymphocytes was calculated by dividing the proportion of Th17 cells by the proportion of Treg cells. Ileum lymphocytes were stimulated with concanavalin A (ConA) **(G)**, and the stimulation index (SI) was calculated as described in the Materials and Methods section. The mRNA levels of *TLR2* **(H)**, *TLR4* **(I)**, *NF-κB* **(J)**, *TNF-α* **(K)**, *IL-4* **(L)**, *IL-6* **(M)**, *IL-10* **(N)**, *IL-17F* **(O)**, *IL-17F/IL-10* **(P)**, *IL-22* **(Q)**, *IFN-γ* **(R)**, *RORα* **(S)**, *Foxp3* **(T)**, *chCD25* **(U)**, *TGF-β1* **(V)**, *NLRP3* **(V)**, *TRIF* **(W)**, *IRAK4* **(W)**, *AhR* **(X)** and *Caspase1* **(X)** in jejunum and ileum were analyzed by RT-PCR. All graphs are presented as mean, with the standard deviation (SD) shown *via* the whiskers. The main effect and interaction effects were analyzed using the general linear model (GLM) procedure, with the *P* values for the main effects written out below each plot. The one-way ANOVA and multiple comparisons were performed when interactive effects differed significantly. The lowercase letters on the bar charts indicate significant differences (*P <*0.05). CON, control group; APS, APS group; NE, NE infection group; NEAPS, APS infected with NE group.

As shown in **Figure 6**, compared with the control group, NE-challenged broilers significantly increased the proportions of d 25 ileum Tc and Treg cells and the proportion of d 31 Th17/Treg cells (*P* < 0.05) and significantly reduced the proportion of d 25 Th cells (*P* < 0.05). There was a tendency to increase the proportion of d 25 Th17 cells (0.05 < *P* < 0.1). Compared with the control group, dietary APS supplementation significantly reduced the proportion of Th17 cells in the ileum at d 25 (*P* < 0.05) and significantly increased the proportion of Tc and Treg cells in the ileum at d 31 (*P* < 0.05). There was a tendency to decrease Th17/Treg cells in the ileum at d 25 (0.05 <*P <*0.1). Compared with unchallenged birds, NE-challenged birds had significantly reduced proliferation activity of T lymphocytes in the ileum at d 25 (*P* < 0.05) at d 25. Compared with the control group, dietary APS supplementation improved the proliferation activity of T lymphocytes in the ileum at d 25 (*P* < 0.05). Compared with the unchallenged birds, NE-challenged birds had significantly increased *TLR2, IRAK4, NF-κB, IFN-γ, TNF-α, TGF-β1, IL-6, IL-10, RORα and Foxp3* mRNA levels (*P* < 0.05) in jejunum on d 31. There was a tendency to increase *IL-17F and IL-17F/IL-10* expression (0.05 < *P* < 0.1) in jejunum. Compared with the control group, dietary APS supplementation significantly reduced the mRNA levels of *TNF-α, IL-17F, and Foxp3* (*P* < 0.05) in jejunum. There was a tendency to downregulate *IL-6 and RORα* (0.05 <*P <*0.1) in jejunum. A strong interaction between dietary APS and NE infection was observed in relation to *TLR2, IFN-γ, TNF-α, IL-6, IL-10, RORα and Foxp3* (*P* < 0.05) in jejunum. Compared with the unchallenged birds, NE-challenged birds had significantly increased *NLRP3, TLR2, IRAK4, NF-κB and IFN-γ* mRNA levels (*P* < 0.05) in ileum on d 31. There was a tendency to increase *RORα* (0.05 < *P* < 0.1) in ileum. Compared with the control group, dietary APS supplementation significantly reduced mRNA levels of *IL-4* and *IL-17F/IL-10* (*P* < 0.05) and significantly increased *AhR and ZO-1* mRNA levels (*P* < 0.05) in ileum on d 31. There was a tendency to increase *Foxp3* and decrease *IL-17F* (0.05 < *P* < 0.1) in ileum. A strong interaction between dietary APS and NE infection was observed in relation to *IFN-γ*, *IL-17F* and *IL-22* (*P* < 0.05) in ileum.

### Ileum Microbiota

#### β Diversity

**Figure 7:**
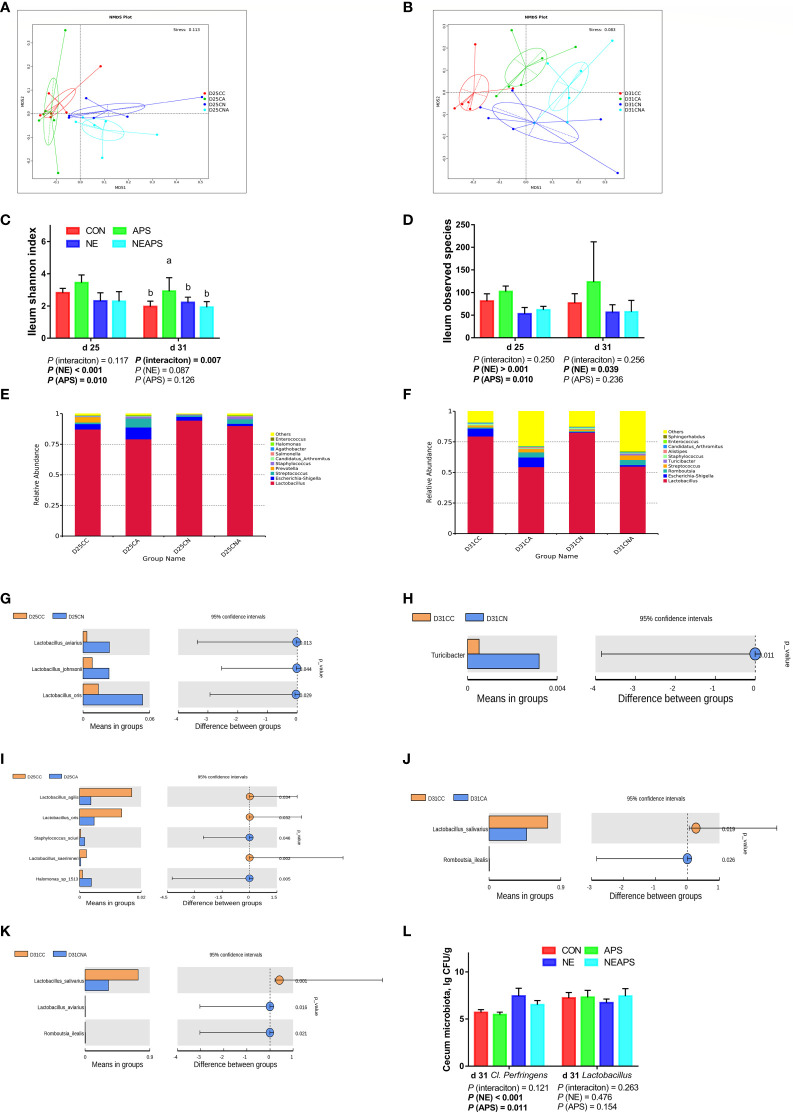
Effects of dietary supplementation of astragalus polysaccharides on the gut microbiota of broiler chickens challenged with necrotic enteritis. The beta diversity **(A, B)** of ileum microbiota of broiler chickens was analyzed by non-metric multidimensional scaling (NMDS). The alpha diversity of ileum microbiota of broiler chickens was analyzed by shannon index **(C)** and observed species **(D)**. Top ten microbiota at the genus level in ileum of broiler chickens on d 25 **(E)** and d 31**(F)**. Differential microbes in ileum of broiler chickens by T-test **(G–K)**. The numbers of *Clostridium perfringens* and *Lactobacillus* **(L)** in cecum were detected by culture count method. All graphs are presented as mean, with the standard deviation (SD) shown *via* the whiskers. The main effect and interaction effects were analyzed using the general linear model (GLM) procedure, with the *P* values for the main effects written out below each plot. The one-way ANOVA and multiple comparisons were performed when interactive effects differed significantly. The lowercase letters on the bar charts indicate significant differences (*P <*0.05). CC/CON, control group; CA/APS, APS group; CN/NE, NE infection group; CNA/NEAPS, APS infected with NE group.

As shown in **Figures 7A, B**, the four groups of CC, CA, CN and CNA on d 25 and d 31 could be separated well, demonstrating that the addition of APS in broiler diets on d 25 and d 31 and infection with necrotic enteritis could cause a significant influence on ileal microbial composition.

#### Alpha Diversity

**Figures 7C, D** shows that compared with the unchallenged birds, NE-challenged birds significantly reduced the observed_species, Shannon, Simpson and Chao1 indices at d 25 and significantly reduced the observed_species and Chao1 indices at d 31 (*P* < 0.05). Dietary APS supplementation significantly increased the observed_species at d 25 (*P* < 0.05). NE and APS supplementation had significant interaction effects on the d 31 Shannon and Simpson indices (*P* < 0.05). Dietary APS supplementation significantly increased the alpha diversity of unchallenged broilers but had no significant effect on NE-challenged broilers.

### Top Ten Microorganisms in the Ileum

**Figures 7E, F** shows that broiler infection with necrotic enteritis increased the relative abundance of d 25 *Lactobacillus* and d 25 *Staphylococcus* and decreased the relative abundance of d 25 *Escherichia-Shigella*, d 25 *Prevotella*, d 25 *Halomonas*, and d 31 *Escherichia-Shigella*.

Dietary APS supplementation increased the relative abundance of d 25 *Escherichia-Shigella*, d 25 *Streptococcus*, d 25 *Staphylococcus*, d 25 *Halomonas*, d 31 *Escherichia-Shigella*, d 31 *Romboutsia* and d 31 *Streptococcus* and decreased the relative abundance of d 25 and d 31 *Lactobacillus*.

### T-Test Test for Different Microorganisms

As shown in **Figures 7G–K**, broiler infection with necrotic enteritis significantly increased d 25 *Lactobacillus* and d 31 *Turicibacter* (*P* < 0.05). The addition of APS to broiler diets significantly increased d 25 *Staphylococcus*, d 25 *Halomonas* and d 31 *Romboutsia* (*P* < 0.05) and significantly reduced d 25 *Lactobacillus* and d 31 *Lactobacillus* (*P* < 0.05).

### Caecal Microbiota

**Figure 7L** shows that compared with the unchallenged birds, NE-challenged birds had significantly increased *Clostridium perfringens* numbers in the caecum at d 31 (*P* < 0.05). Compared with the control group, dietary APS supplementation significantly reduced the number of *Clostridium perfringens* in the caecum at d 31 (*P* < 0.05).

### Non-Targeted Metabolome Analysis of Ileum Contents

#### Data Quality Assessment and Comparison of Differences Between Groups

**Figure 8:**
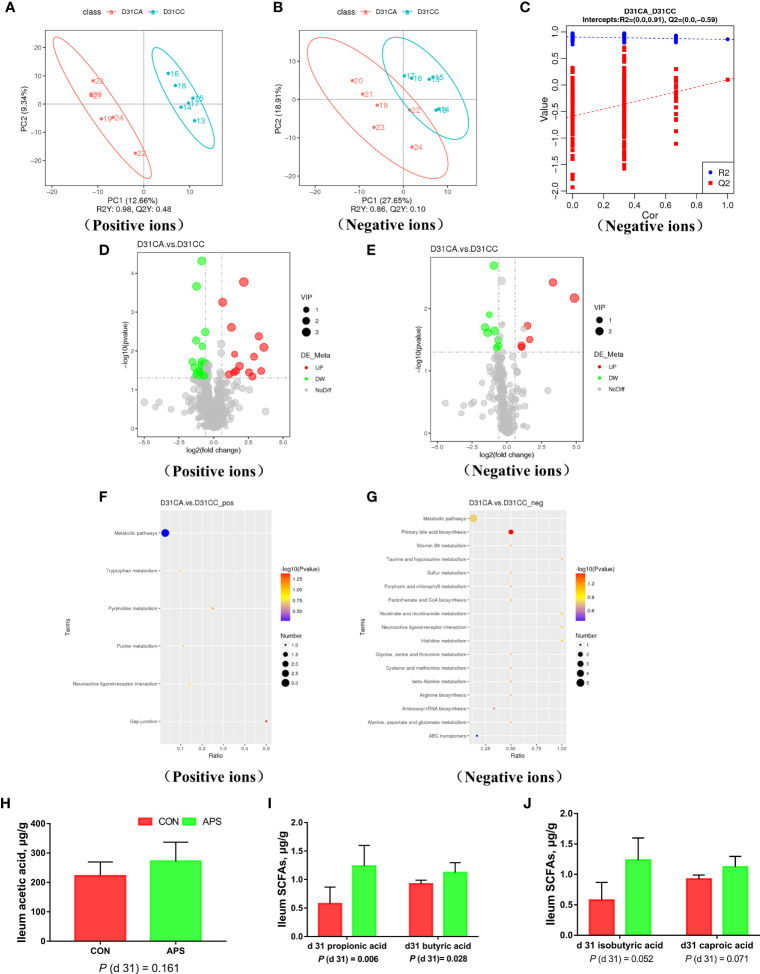
Effects of dietary supplementation of astragalus polysaccharides on ileal metabolome and short-chain fatty acids of broiler chickens. PLS-DA scatter plot for group CA group vs. CC group **(A, B)**. Permutation test **(C)** of PLS-DA model for group CA versus CC (negative ion mode). Volcano plot for group CA versus CC **(D, E)**. Each point in the mountain chart represents a metabolite, the abscissa represents the fold change (take the log 2 logarithm) of each substance in the group, and the ordinate represents the P-value of the T test (take the log 10 logarithm). The size of the scatter points represents the VIP value of the PLS-DA model, and the larger the scatter point, the greater the VIP value. Scattered colors represent the final screening results. Significantly up-regulated metabolites are shown in red, significantly down-regulated metabolites are shown in green, and non-significantly different metabolites are shown in grey. CA group versus CC group KEGG pathway enrichment analysis bubble chart **(F, G)**. According to the results of KEGG enrichment, select the top 20 pathways sorted by P-values from small to large to draw a bubble chart. The abscissa is x/y (the number of differential metabolites in the corresponding metabolic pathway / the total number of metabolites identified in the pathway), the larger the value, the higher the enrichment of differential metabolites in the pathway, and the ordinate is the KEGG pathway name. The ordinate and color of the bubble indicate the P-values of the enrichment analysis (take the negative common logarithm, ie -log 10 P-values). The redder the color, the smaller the P-values, indicating that the degree of enrichment is more significant, the reliability of the test is greater and the difference in statistics is more significant. The size of the dot represents the number of different metabolites in the corresponding pathway. The larger the dot, the more differential metabolites in the pathway. The levels of short-chain fatty acids **(H–J)** in the ileal contents of broiler chickens were analyzed by high performance liquid chromatography (Dionex, USA). All graphs are presented as mean, with the standard deviation (SD) shown via the whiskers. Statistical differences were determined by one-way ANOVA, with the P values for the main effects written out below each plot. P-values represent the effect of the APS. CA, dietary APS supplementation group; CC, control group.

To ensure the reliability of the data, the influence of factors such as the sample preparation process and instrument instability must be minimized. In the process of detection and analysis, three QC samples are used to monitor and ensure the stability of the instrument, after which the missing values are simulated in the original data to standardize the data. After obtaining the sorted data, we perform partial least squares discrimination analysis (PLS-DA). Orthogonal partial least squares-discriminant analysis was performed on the data, and the PLS-DA model of the APS supplementation group versus the control group was obtained. As shown in **Figures 8A, B**, compared with the control group CC, in the CA ileum content of the APS supplementation group, the middle metabolites changed significantly, and there were significant differences between the two treatment groups. The results of the permutation test of the PLS-DA model are shown in **Figure 8C** (intercept R2 = 0.91, Q2 = -0.59). Thus, it can be seen that the PLS-DA model does not have an overfitting phenomenon, and the model has good robustness.

### Differential Metabolite Screening Between Groups

According to the relative molecular mass and the secondary mass spectrometry data combined with the HMDB, PubChem and KEGG databases, a comparison search was conducted to analyze the potential differential metabolites. According to the condition that the variable importance in the projection (VIP) of the first principal component of the PLS-DA model was greater than 1 and *P* < 0.05, 35 different metabolites were identified in positive ion mode (**Table 3**). There were 14 types in negative ion mode (**Table 4**). Compared with the blank control group, the metabolites that were increased in the ileal content of broiler diets with APS were mainly formononetin, bile acid, taurine and bile acid intermediates, equol, glycitein and 20 other kinds, while serotonin (5-HT), uric acid, L-arginine, DL-lysine, indole-derived salt, cystine, 4-pyruvate, L-tianmen 29 kinds of aspartic acid, thymine, and protoporphyrin IX were downregulated. As shown in **Figures 8D, E**, the screening of differential metabolites was visualized in the form of a volcano map.

**Table 3:** Up-regulated and down-regulated metabolites in ileum from group CA^a^ vs CC^b^ (Positive ions).

Items	Name	Fold Change	*P*-values^c^	VIP^d^	Molecular Weight
Up-regulated metabolites					
1	Tetranor-12(S)-HETE	4.52	<0.001	3.62	271.1
2	N4-(2,6-dimethoxy-3-pyridyl)-3,5-dimethyl-4-isoxazolesulfonamide	1.57	<0.001	2.94	313.1
3	4-[2-(4-chlorophenyl) diaz-1-enyl]-2-methyl-6-(piperidinomethyl)phenol	2.44	<0.001	2.85	343.1
4	MAG (18:3)	9.54	<0.001	2.28	352.3
5	(S)-Equol	12.29	0.010	3.00	242.1
6	N-(1,3-benzodioxol-5-ylmethyl)-6-morpholinonicotinamide	2.85	0.010	1.35	341.1
7	Jervine	7.48	0.010	2.03	425.3
8	Sodium cholate	3.65	0.020	2.67	430.3
9	FMH	10.77	0.030	1.99	433.2
10	Formononetin	3.07	0.030	2.02	268.1
11	Glycitein	2.75	0.040	1.87	284.1
12	Milbemycin A4 oxime	5.87	0.040	2.14	555.3
13	diethyl 2-[(4-benzhydrylpiperidino) methylidene] malonate	2.15	0.040	2.19	443.2
14	7-Ketodeoxycholic acid	6.92	0.050	2.04	406.3
Down-regulated metabolites					
1	EMH	0.55	<0.001	2.97	397.1
2	Uric Acid	0.43	<0.001	2.84	168.0
3	Taurocholic acid sodium salt hydrate	0.66	<0.001	2.36	538.3
4	NPK	0.42	0.010	2.44	714.4
5	Thymine	0.57	0.010	1.81	126.0
6	L-arginine	0.56	0.020	2.68	174.1
7	15-Deoxy-Δ12,14-prostaglandin J2-2-glycerol ester	0.35	0.020	2.16	408.2
8	Serotonin	0.65	0.020	2.41	176.1
9	FRH	0.55	0.020	2.43	480.2
10	ACar 10:1	0.39	0.030	2.42	313.2
11	1,3-diazaspiro [4.5] decane-2,4-dione	0.58	0.030	2.12	168.1
12	LPC 22:6	0.62	0.030	1.60	567.3
13	DL-Lysine	0.59	0.030	2.09	129.1
14	5,6-diphenyl-2,3-dihydropyrazine	0.50	0.030	2.11	234.1
15	PC (18:5e/2:0)	0.43	0.040	1.78	541.3
16	2-Hydroxycinnamic acid	0.54	0.040	1.94	164.0
17	PC (18:4e/2:0)	0.42	0.040	1.90	543.3
18	Gly-Val	0.63	0.040	2.11	174.1
19	Loxoprofen	0.62	0.040	2.22	246.1
20	2-Hydroxyphenylalanine	0.52	0.050	1.87	181.1
21	3-Methylcrotonylglycine	0.64	0.050	1.72	140.0

a CA, astragalus polysaccharide group;

b CC, control group;

c P-values represent the effect of the APS;

d VIP, variable importance in projection.

**Table 4:** Up-regulated and down-regulated metabolites in ileum from group CA^a^ vs CC^b^ (Negative ions).

Items	Name	Fold Change	*P*-values^c^	VIP^d^	Molecular Weight
Up-regulated metabolites					
1	Cholic acid	9.98	<0.001	1.91	408.3
2	Equol	29.12	0.010	2.86	242.1
3	Glycoursodeoxycholic acid	2.80	0.020	1.49	449.3
4	Cholesteryl sulfate	3.13	0.030	1.32	466.3
5	Taurine	2.08	0.040	1.75	125.0
6	Dihydroroseoside	2.08	0.040	1.34	388.2
Down-regulated metabolites					
1	DL-4-Hydroxyphenyllactic acid	0.53	<0.001	2.19	182.1
2	Protoporphyrin IX	0.42	0.010	1.05	562.3
3	Cystine	0.34	0.020	1.89	240.0
4	4-Pyridoxic acid	0.54	0.020	2.47	183.1
5	Mestranol	0.38	0.020	2.24	310.2
6	N-Acetylalanine	0.65	0.030	1.75	131.1
7	L-Aspartic acid	0.66	0.040	1.60	133.0
8	3-Indoxyl sulphate	0.60	0.040	1.55	213.0

a CA, astragalus polysaccharide group;

b CC, control group;

c P-values represent the effect of the APS;

d VIP, variable importance in projection.

### Differential Metabolite KEGG Analysis

**Table 5** shows that the differential metabolites between groups were mapped to 23 metabolic pathways in the KEGG database: gap junction, pyrimidine metabolism, neuroactive ligand-receptor interaction, purine metabolism, tryptophan metabolism, metabolic pathways, primary bile acid biosynthesis, histidine metabolism, taurine and hypotaurine metabolism, nicotinamide and nicotinamide metabolism, neuroactive ligand-receptor interaction, metabolic pathways, arginine biosynthesis, alanine, aspartate and glutamate metabolism, glycine, serine and threonine metabolism, cysteine and methionine metabolism, beta-alanine metabolism, vitamin B6 metabolism, vitamin B6 metabolism pantothenate and CoA biosynthesis, porphyrin and chlorophyll metabolism, sulfur metabolism, aminoacyl-tRNA biosynthesis, and ABC transporters.

**Table 5:** Annotation of KEGG^a^ pathways.

Items	Map Title	*P*-values^b^	Meta IDs
Positive ions			
1	Gap junction	0.04	Serotonin
2	Pyrimidine metabolism	0.08	Thymine
3	Neuroactive ligand-receptor interaction	0.14	Serotonin
4	Purine metabolism	0.17	Uric Acid
5	Tryptophan metabolism	0.19	Serotonin
6	Metabolic pathways	0.56	Thymine, Serotonin, Uric Acid
Negative ions			
1	Primary bile acid biosynthesis	0.04	Taurine, Cholic acid
2	Histidine metabolism	0.09	L-Aspartic acid
3	Taurine and hypotaurine metabolism	0.09	Taurine
4	Nicotinate and nicotinamide metabolism	0.09	L-Aspartic acid
5	Neuroactive ligand-receptor interaction	0.09	Taurine
6	Metabolic pathways	0.16	Taurine, Protoporphyrin IX, 4-Pyridoxic acid, Cholic acid, L-Aspartic acid
7	Arginine biosynthesis	0.17	L-Aspartic acid
8	Alanine, aspartate and glutamate metabolism	0.17	L-Aspartic acid
9	Glycine, serine and threonine metabolism	0.17	L-Aspartic acid
10	Cysteine and methionine metabolism	0.17	L-Aspartic acid
11	beta-Alanine metabolism	0.17	L-Aspartic acid
12	Vitamin B6 metabolism	0.17	4-Pyridoxic acid
13	Pantothenate and CoA biosynthesis	0.17	L-Aspartic acid
14	Porphyrin and chlorophyll metabolism	0.17	Protoporphyrin IX
15	Sulfur metabolism	0.17	Taurine
16	Aminoacyl-tRNA biosynthesis	0.25	L-Aspartic acid
17	ABC transporters	0.45	Taurine

The order of enriched pathways was displayed based on hits and pathway impact. * Only those pathways with hits > 2 and adjust P-values < 0.05 were considered significantly enriched.

a KEGG, kyoto encyclopedia of genes and genomes;

b P-values represent the effect of the APS.

### Enrichment Analysis of Metabolic Pathways of Differential Metabolites

The metabolites of differences between groups were entered into MetaboAnalyst, the database corresponding to chickens was used to perform enrichment analysis on metabolic pathways, and the corresponding P values were calculated. Through topological and enrichment analyses of the metabolic pathways where the differential metabolites are located, the metabolic pathways are screened in depth to identify the metabolic pathways that are more relevant to the experimental treatment. The screening conditions were *P* < 0.05 in the enrichment analysis and an influence value greater than 0.1 in the topological analysis. As shown in **Figures 8F, G**, through enrichment analysis of differential metabolites combined with topological analysis, the metabolic pathways significantly enhanced by APS supplementation mainly included primary bile acid biosynthesis, tryptophan metabolism, histidine metabolism, taurine and hypotaurine metabolism, nicotinate and nicotinamide metabolism, gap junctions, neuroactive ligand-receptor interactions, pyrimidine metabolism and purine metabolism.

### Short-Chain Fatty Acids in Ileal Contents

**Figures 8H–J** shows that compared with the blank control group, the addition of APS to the broiler diet significantly increased the levels of propionic acid and butyric acid in the ileal content (*P* < 0.05). There was a tendency to increase isobutyric acid and caproic acid (0.05 < *P <*0.1).

### Correlation Heat Map

**Figure 9:**
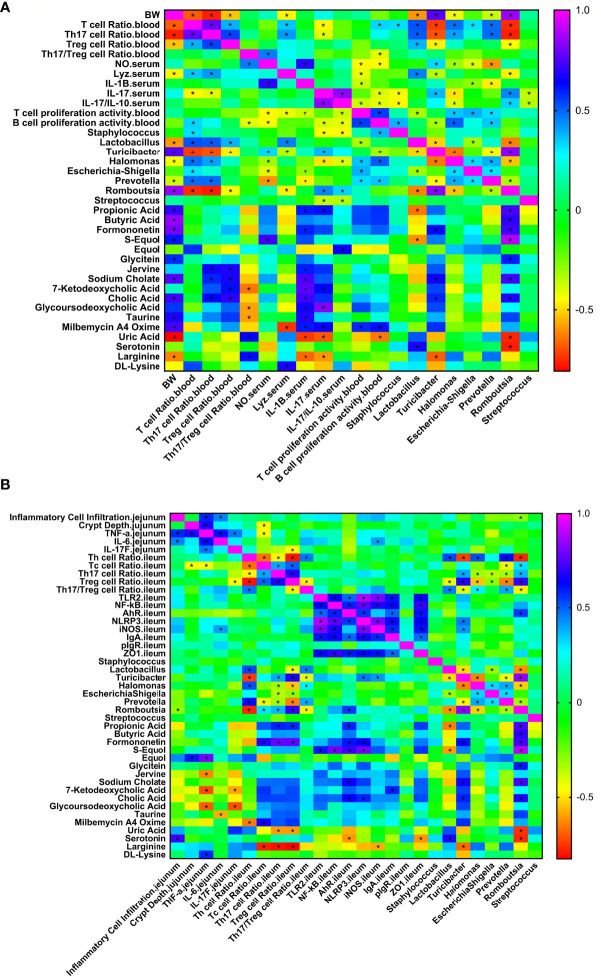
Correlation heat map of ileum differential bacteria and differential immune function of broiler chickens. Spearman’s correlations were calculated for all significantly different peripheral **(A)** and intestinal **(B)** immune indexes and different ileal microbes on genus level. Colors of squares represent r values of spearman’s correlation coefficient. *P < 0.05.

To explore the relationships between the intestinal microbiota, intestinal metabolites and apparent immune indicators in broiler chickens, based on the abovementioned ileal microbiome and metabolomics data, we subsequently performed a spearman correlation analysis (**Figure 9**) to determine the microbes and metabolites related to the immunomodulatory effect of APS.

As shown in **Figure 9A**, production performance was positively correlated with *Turicibacter*, *Romboutsia*, propionic acid, butyric acid, formononetin, S-equol, and cholic acid and was negatively correlated with *Lactobacillus*, *Halomonas*, *Prevotella*, uric acid, and L-arginine (*P* < 0.05). The proportion of Th17 cells in peripheral blood was positively correlated with *Lactobacillus*, *Halomonas*, *Prevotella*, Jerrivne, sodium cholate and cholic acid and negatively correlated with *Turicibacter* and *Romboutsia* (*P* < 0.05). The proportion of Treg cells in peripheral blood was positively correlated with *Lactobacillus*, jervine, sodium cholate, 7-ketodeoxycholic acid and cholic acid and negatively correlated with *Turicibacter* and *Romboutsia* (*P* < 0.05). The peripheral blood Th17/Treg ratio was positively correlated with uric acid and L-arginine (*P* < 0.05) and negatively correlated with 7-ketodeoxycholic acid, glycoursodeoxycholic acid and taurine (*P* < 0.05). Serum IL-1β was positively correlated with propionic acid, formononetin, jervine, sodium cholate, 7-ketodeoxycholic acid, cholic acid, glycoursodeoxycholic acid, taurine and milbemycin A4 oxime (*P* < 0.05) and negatively correlated with *Escherichia-Shigella*, *Prevotella*, uric acid and L-arginine (*P* < 0.05). Serum IL-17 was positively correlated with *Turicibacter*, *Romboutsia*, propionic acid, 7-ketodeoxycholic acid, glycoursodeoxycholic acid and milbemycin A4 oxime (*P* < 0.05) and negatively correlated with *Staphylococcus*, *Halomonas*, *Streptococcus*, uric acid and L-arginine (*P* < 0.05). Serum IL-17/IL-10 was positively correlated with *Romboutsia* and equol (*P* < 0.05) and negatively correlated with *Staphylococcus*, *Halomonas* and *Streptococcus* (*P* < 0.05). Peripheral blood T cell proliferation activity was positively correlated with *Halomonas*, *Escherichia-Shigella*, *Prevotella* and milbemycin A4 oxime (*P* < 0.05) and negatively correlated with *Lactobacillus* (*P* < 0.05). Peripheral blood B cell proliferation activity was positively correlated with *Halomonas*, *Prevotella* and milbemycin A4 oxime (*P* < 0.05) and negatively correlated with *Turicibacter* and uric acid (*P* < 0.05). *Lactobacillus* was positively correlated with serotonin and negatively correlated with propionic acid and S-equol (*P* < 0.05). As shown in **Figure 9B**, jejunal inflammatory cell infiltration was negatively correlated with *Romboutsia* (*P* < 0.05). The depth of jejunal crypts was positively correlated with equol (*P* < 0.05). Jejunal *TNF-α* gene expression was positively correlated with equol and DL-lysine and negatively correlated with jervine, 7-ketodeoxycholic acid and glycoursodeoxycholic acid (*P* < 0.05). The expression of the *IL-6* gene in the jejunum was negatively correlated with taurine (*P* < 0.05). The expression of the *IL-17F* gene in the jejunum was negatively correlated with 7-ketodeoxycholic acid and glycoursodeoxycholic acid (*P* < 0.05). The proportion of Th cells in the ileum was positively correlated with *Lactobacillus*, *Halomonas* and *Prevotella* (*P* < 0.05) and negatively correlated with *Turicibacter*, *Romboutsia* and milbemycin A4 oxime (*P* < 0.05). The proportion of Tc cells in the ileum was positively correlated with *Romboutsia*, formononetin and milbemycin A4 oxime (*P* < 0.05) and negatively correlated with *Prevotella* and L-arginine (*P* < 0.05). The proportion of Th17 cells in the ileum was positively correlated with *Turicibacter*, *Romboutsia*, propionic acid and formononetin (*P* < 0.05) and negatively correlated with *Halomonas*, *Escherichia-Shigella*, *Prevotella*, uric acid and L-arginine (*P* < 0.05). The proportion of Treg cells in the ileum was positively correlated with *Turicibacter*, *Romboutsia*, propionic acid and formononetin (*P* < 0.05) and negatively correlated with *Lactobacillus*, *Halomonas*, *Escherichia-Shigella*, *Prevotella*, uric acid and L-arginine (*P* < 0.05). The ratio of Th17/Treg cells in the ileum was positively correlated with *Lactobacillus*, *Halomonas*, and *Prevotella* (*P* < 0.05) and negatively correlated with *Turicibacter* and *Romboutsia* (*P* < 0.05). The expression of *TLR2* and *NF-κB* genes in the ileum was positively correlated with S-equol (*P* < 0.05). The expression of the *iNOS* gene in the ileum was positively correlated with *Turicibacter* and negatively correlated with L-arginine (*P* < 0.05). *IgA* gene expression in the ileum was positively correlated with 7-ketodeoxycholic acid (*P* < 0.05). Ileal *ZO-1* gene expression was negatively correlated with serotonin (*P* < 0.05). In addition, *Lactobacillus* was positively correlated with serotonin and negatively correlated with propionic acid and S-equol (*P* < 0.05). *Turicibacter* was positively correlated with formononetin, sodium cholate and cholic acid (*P* < 0.05) and negatively correlated with L-arginine (*P* < 0.05). *Romboutsia* was positively correlated with propionic acid, butyric acid, formononetin, S-equol, glycitein, sodium cholate and cholic acid (*P* < 0.05) and negatively correlated with uric acid and serotonin (*P* < 0.05). The results show that the immunomodulatory effect of APS is associated with a specifically altered microbe-metabolic axis.

## Discussion

Necrotic enteritis (NE) is an enterotoxic disease caused by *Clostridium perfringens*. This disease often occurs in broilers at 2–5 weeks of age, with a mortality rate of approximately 2%-10%, with a maximum of 50%. Geier et al. (30) showed that necrotic enteritis can increase the broiler feed-to-meat ratio, mortality and intestinal disease and reduce body weight. The main reason for the decline in growth performance is that coccidia cause proteins (including plasma) to leak into the intestinal lumen, which promotes the rapid reproduction and pathogenicity of toxin-producing *Clostridium perfringens*, resulting in a decrease in the digestion and absorption capacity of the small intestine (1). It is estimated that the poultry industry loses up to US$2 billion each year (2). Consistent with previous studies (17, 31), this experiment also observed that NE infection has a significant negative impact on broiler body weight and body weight gain. Supplementation with APS can effectively improve the decline in growth performance caused by NE infection. APS improves the growth performance of chickens (32, 33) but has a positive effect on animal growth in other animals, such as pigs (34, 35). APS alleviates the decline in growth performance caused by NE, which may be due to the direct bacteriostasis of APS, which reduces the number of *Clostridium perfringens*, or it may regulate the host immune function to sterilize and achieve indirect bacteriostasis, thereby reducing intestinal damage and the loss of growth performance. Therefore, the next step is to test the immune function of broilers.

First, we tested the systemic immune response and found that NE infection reduced the broiler thymus index, increased peripheral blood NO levels and lysozyme activity, increased the Th17 cell ratio and Th17/Treg cell ratio, and increased the production of the proinflammatory cytokines IL-17 and Th17/IL-10. At the same time, other inflammatory cytokines, such as IL-1β and IFN-γ, and the anti-inflammatory cytokine IL-4 were increased, reducing the proliferation capacities of T and B lymphocytes in the preinfection period, indicating that NE Infection of broilers caused systemic inflammation and simultaneously suppressed systemic cellular immune function. Previous studies have found that NE-challenged broilers significantly upregulate the expression of splenic inflammatory cytokine genes, causing systemic inflammation (36, 37). It has been reported that enterotoxigenic E. coli-challenged mice significantly upregulate the proportions of Th17 and Treg cells in the spleen and intestinal mucosal lymph nodes, upregulate the mRNA and protein expression of RORα in the intestinal mucosal lymph nodes, and downregulate the mRNA and protein expression of Foxp3 (4). Another study found that coccidia infection significantly increased the proportion of Th17 cells in the spleen and caecal tonsils (6), which is consistent with the findings of this study. We speculate that the reason for the more serious systemic inflammatory response caused by NE may be that strong intestinal inflammation leads to damage to the intestinal barrier. Pathogens and proinflammatory cytokines enter the blood through intestinal epithelial cells, stimulate peripheral immune organs and cause systemic inflammation. Dietary APS supplementation can increase the ratio of Treg cells and the IL-10 levels in peripheral blood, reduce the ratio of Th17/Treg cells and Th17/IL-10 cytokines, and alleviate the systemic inflammation caused by NE. Liu et al. found that APS can alleviate the immune stress response of chickens induced by LPS mainly by reducing the transcription of genes such as TLR4 and NF-κB (38). In a mouse study, it was found that intraperitoneal injection of APS downregulated the ratio of Th2 and Th17 cells in the peripheral blood of mice challenged with bacterial sepsis (39), consistent with the results of this study, indicating that dietary APS supplementation can alleviate the systemic inflammation caused by NE. We speculate that this may be due to APS alleviating intestinal inflammation and improving the intestinal barrier, thereby indirectly alleviating systemic inflammation.

The more serious systemic inflammatory response in broilers after NE infection may be related to intestinal inflammation and the impaired intestinal barrier (40). The damage to the intestinal tract is caused by pathogenic bacteria and dietary supplemented APS. As the intestine is the first target organ of action, we also need to pay attention to the situation of intestinal inflammation. In view of the characteristics of NE infection, intestinal health and function were also the focus of this trial. The scores of intestinal lesions, histopathology scores, intestinal tissue morphology and the number of caecal pathogens are important indicators for assessing intestinal health, functional integrity and disease recovery. In this trial, NE infection caused bleeding points in the jejunum and ileum, and the degree of jejunum lesions was stronger than that of the ileum. The score of intestinal lesions, histopathological scores of the jejunum and the number of *Clostridium perfringens* in the caecum were significantly increased, and the structure of the jejunum was severely damaged. The decrease in sIgA in the ileal mucosa indicated that the NE model was successfully established, the intestinal mucosa had a strong inflammatory response and some mucosal immune functions were impaired. These results are consistent with previous studies, indicating that NE infection causes inflammation of the intestinal mucosa inflammatory response and simultaneously damages the intestinal barrier structure and increases the permeability of the intestine. Pathogens and proinflammatory cytokines pass through the intestinal barrier and into the blood, which induces the occurrence of systemic inflammation (17, 31, 41). However, dietary APS supplementation reduced intestinal lesion scores and pathological scores, increased jejunum V/C values, and reduced crypt depth. Therefore, we inferred that dietary APS supplementation alleviated the intestinal damage caused by NE and prevented pathogenic bacteria from entering the bloodstream, which alleviated the systemic immune response.

To clarify the mechanism of dietary APS supplementation in intestinal inflammation and intestinal health, we further evaluated the ratio and proliferation activity of Th17/Treg cells in the ileum and the changes in the expression profiles of related immune signalling pathways and related downstream target genes in the intestine. Th17 and Treg cells in the body are related to each other developmentally, and they transform into each other under the control of a variety of factors. Th17 cells play an important role in the development of autoimmunity and inflammation and are key in the immune response; Treg cells regulate the systemic immune response by regulating the activity of effector T cells and maintaining peripheral immune tolerance. The transcription factors Foxp3+ and RORγt are specific transcription factors of Treg cells and Th17 cells, respectively, and are closely related to the differentiation of the two. Regulating the balance of Foxp3+ and RORγt can indirectly correct the imbalance of Th17/Treg cells. The results of this study found that NE infection resulted in the upregulation of the ratios of Tc and Th17 cells and Th17/Treg cells in the ileum, the decrease of the ratio of Tc cells, the activation of TLR2-NF-κB signals in the jejunum and ileum, and upregulation of the expression of the Th17 cell-related genes RORα, IL-6, TGF- β1. These results indicate that NE infection differentially regulates the expression of intestinal immune-related genes, and the Th17/Treg cell ratio is unbalanced, leading to aggravation of the intestinal immune inflammatory response. Studies have found that large amounts of STAT-3, IL-1β, IL-6 and IL-17 are produced in caecal intraepithelial lymphocytes in broiler chickens challenged by *E. coli* (42). Coccidia infection in broilers significantly increases the proportions of Th17 cells in the spleen and caecal tonsils (6). Previous studies on mouse colitis have found that IL-1β is significantly increased when intestinal inflammation occurs, which mediates chronic intestinal inflammation by promoting the accumulation of IL-17A-secreting innate lymphoid cells and Th17 cells (3). It has been reported that enterotoxigenic *E. coli*-challenged mice significantly upregulate the proportion of Th17 and Treg cells in the spleen and intestinal mucosal lymph nodes, upregulate the mRNA and protein expression of RORα in the intestinal mucosal lymph nodes, and downregulate the mRNA and protein expression of Foxp3 (4). Other studies have found that TNBS-induced colitis and DSS-induced colitis show significant increases in intestinal IL-17 (5), consistent with the findings of this study. Dietary APS supplementation can decrease intestinal RORα, IL-17F, and IL-17F/IL-10 levels, increase ileal IL-4 mRNA levels, reduce the ileal Th17 and Th17/Treg cell ratios, increase ileal T and B cell proliferation activity, increase the expression of the IL-4 gene in the ileum, and simultaneously increase ZO-1 to improve the damage to the intestinal barrier caused by NE, reduce the intestinal lesion score and pathological score, and alleviate the intestinal inflammation caused by NE. Other scholars have pointed out that the addition of APS can reduce the overexpression of proinflammatory cytokines caused by LPS by inhibiting the TLR-NF-κB signalling pathway (43). Studies have also found that dietary supplementation with 400 ppm APS can significantly reduce IL-17 levels and RORα expression in the colon of mice with TNBS-induced colitis and increase the proportion of Treg cells in the colon (12), consistent with the results of this study. Therefore, these results demonstrate that the addition of APS not only enhances the body’s innate immune defence response to pathogen infection by regulating the TLR signalling pathway but also balances the proinflammatory/anti-inflammatory responses of intestinal Th17/Treg cells to avoid excessive inflammation and maintain immune homeostasis. This effect is achieved by downregulating the proportions of proinflammatory Th17 cells and their cytokines and upregulating the expression levels of anti-inflammatory Tregs and their cytokines.

According to the scores of intestinal lesions and pathological scores, ileal lesions are higher than those in the jejunum, and the abundance of microorganisms in the ileum of broilers is significantly higher than that in the jejunum (44); therefore, the interaction effect between microorganisms and the host may be more important in the ileum. strong. Combined with previous research reports, the segmented filamentous bacteria *Helicobacter* and *Clostridium* and the SCFAs produced by intestinal microbes can regulate the differentiation of intestinal Th17 and Treg cells (13). This study also found that dietary APS can increase the abundance of segmented filamentous bacteria and *Romboutsia* that can produce SCFAs. To clarify whether APS can regulate mucosal immune function and alleviate mucosal immune function by improving the gut microbiota through the mechanism of intestinal inflammation, we evaluated the microbiota, SCFAs and metabolome of ileum. We found that NE infection in broilers significantly increased the number of *Clostridium perfringens* in the caecum and the relative abundance of *Lactobacillus*, *Staphylococcus* and *Turicibacter* in the ileum. *Lactobacillus* is mostly beneficial bacteria, and NE infection increases its abundance. We speculate that the reason may be that *Lactobacillus* itself is the dominant ileum genus, and the abundance remains above 80%, while NE infection leads to the living environment of intestinal microbes. The changes in the ileum reduce the diversity of ileal microbial species α, which is specifically reflected in the reduction of the abundance of *Escherichia-Shigella*, the second most common bacterium in the control group, and provides a living space for the increase in the abundance of *Lactobacillus*. Regarding *Staphylococcus*, studies have found that most species pathogenic bacteria, among which *Staphylococcus aureus* can cause serious animal diseases, such as mastitis, purulent disease, arthritis and urinary tract infections (45, 46). It has been reported that the abundance of *Turicibacter* in the colon of mice with acute dextran sulfate (DSS)-induced colitis increases and is significantly positively correlated with DSS treatment (47), which is consistent with the findings of this study. These findings indicate that NE infection can reduce the ileal microbial alpha diversity and increase the abundance of the harmful bacteria *Staphylococcus* and *Turicibacter* associated with DSS enteritis. However, dietary APS supplementation significantly reduced the number of *Clostridium perfringens* in the caecum, significantly increased the abundance of *Romboutsia*, *Staphylococcus*, and *Halomonas* in the ileum, and decreased the abundance of *Lactobacillus*. According to reports, *Romboutsia* is a gram-positive coccus that is more common in healthy human mucosa and may be related to host health (48). *Romboutsia* mainly uses monosaccharides and disaccharides to form acetic acid, formic acid and lactic acid (49). *Lactobacillus* is the dominant genus of the ileum, and the increase in the abundance of other microorganisms will inevitably reduce its abundance. The results showed that APS supplementation improved the ileal microbiota and increased the abundance of the SCFA-producing bacteria *Romboutsia*. Therefore, the level of microbial metabolite SCFAs in the ileum may also increase.

Changes in microbiota lead to changes in microbes and host metabolism. By targeting the metabolome, we found that APS supplementation significantly increased the levels of propionic acid and butyric acid in the ileal contents of d 31 broilers, and there was a tendency to increase isobutyric acid and caproic acid levels. Short-chain fatty acids (SCFAs) play an important role in intestinal physiology. According to reports, SCFAs, especially butyrate, can be used as an energy source for colonic epithelial cells (50). In addition, SCFAs have a negative impact on the expression of virulence factors of bacterial pathogens (51). Studies have also found that in mice, the SCFA butyrate produced by the fermentation of symbiotic microorganisms in the starch in the back half of the intestine helps the thymus to produce Treg cells, and the produced propionic acid can inhibit histone deacetylase. (HDAC), which enhances the production of new Treg cells in peripheral blood (52), consistent with the results in the peripheral blood and ileum in this study. The results indicated that dietary APS supplementation may increase the levels of propionic acid and butyric acid by increasing the abundance of *Romboutsia* in the ileum, protecting intestinal epithelial cells, and increasing the proportion of Treg cells in the peripheral blood to alleviate inflammatory damage caused by NE.

We measured the nontarget metabolome of the ileal content and found that dietary APS supplementation increased formononetin, bile acid, taurine and bile acid intermediates, equol, and glycitein and reduced 5-HT, uric acid, an indole-derived salts in the ileal content. Through enrichment analysis of differential metabolites combined with topological analysis, the metabolic pathways significantly enhanced by APS supplementation mainly included primary bile acid biosynthesis, tryptophan metabolism, histidine metabolism, taurine metabolism, nicotinate and nicotinamide metabolism, gap junctions, neuroactive ligand-receptor interactions, pyrimidine metabolism and purine metabolism. Previous studies have found that formononetin is a flavonoid compound that has anti-inflammatory, antioxidant, lipid metabolism, and antitumor activities in Astragalus, Spatholobi and other plants (53). These results indicate that in addition to directly regulating the immune function of the host, APS may also be metabolized by intestinal microorganisms to produce formononetin to regulate the mucosal immune function of broilers. Studies have also found that intraperitoneal injection of formononetin can slow DSS-induced colitis by inhibiting NLRP3 inflammasomes (54). It has been reported that bile acids can promote the differentiation and activity of Th17 cells and Tregs involved in regulating inflammation and can regulate the differentiation and activity of Th17 cells and Tregs associated with intestinal inflammatory diseases (55, 56). Taurine is a sulfur-containing amino acid in animals that has biological effects such as improving immune function, antioxidation, and lowering blood sugar (57, 58). The composition of murine intestinal commensal bacteria (*Bacteroides polymorpha*, *Bacteroides fragilis*) in the murine intestinal bile acid pool regulates the number of RORγ+ regulatory T cells in the colon. In SPF mice with insufficient diet and nutrition, the intestinal bile acid pool was restored (replenishing a specific combination of primary or secondary bile acids), colon RORγ+ Tregs were increased through the bile acid-VDR axis, and the susceptibility to colitis was reduced (59). S-equol and glycitein are derived from soybeans, have oestrogenic and antiestrogenic properties, and have anti-inflammatory and antioxidant effects (60, 61). Equol is an important product formed by the microbial metabolism of soybean isoflavones, a secondary metabolite formed during the growth of soybeans (62) and possibly a metabolite of the active ingredients of soybean meal produced by ileal microorganisms. Jervine comes from the Veratrum plant and is a steroidal alkaloid. It has anti-inflammatory, antioxidant and antihypertensive effects (63). According to reports, uric acid may be a danger signal released by damaged cells, which can stimulate the NFκB signalling pathway of dendritic cells to promote the release of cytokines related to Th17 differentiation and then induce inflammation and a strong immune response (64). Arginine is an essential amino acid in poultry, and poultry lack carbamoyl phosphate synthase and dihydropyrrole-5-carboxylic acid synthase, which are necessary to synthesize arginine. However, some studies have found that excessive L-arginine may cause inflammatory episodes such as pancreatitis in animals (65, 66). Through spearman correlation analysis, we found that the ratio of Th17/Treg cells was mainly positively correlated with *Lactobacillus*, *Halomonas*, *Prevotella*, uric acid and L-arginine and negatively correlated with *Romboutsia* and *Turicibacter*. *Romboutsia* was positively correlated with propionic acid, butyric acid, formononetin, S-equol, glycitein, sodium cholate and cholic acid and negatively correlated with uric acid and serotonin. Correlation analysis results indicate that *Romboutsia* may upregulate formononetin and equol by producing enzymes to degrade substrates such as APS and soybean meal and downregulate metabolites such as uric acid to regulate Th17/Treg balance and host immune function, thereby producing beneficial effects.

## Conclusion

Taken together, dietary APS supplementation improved the production performance and immune function of broilers challenged with NE and regulated the Th17/Treg balance. Further microbiome and metabolomics studies demonstrated that APS induced structural rearrangement of gut microbiota, mediating alterations in a wide range of metabolites. With clustering and correlation analysis, the immunoregulatory effect of APS was highly correlated with an increased abundance of *Romboutsia*, which was linked to the upregulation and downregulation of a range of metabolites, such as formononetin, propionic acid, butyric acid, sodium cholate, bile acid, S-equol, glycitein, uric acid, and L-arginine. The specific alterations within the microbe-metabolic axis may play an important role in the immunoregulation of APS. The results provide new insights into the regulatory effect of APS on the immune system of broiler chickens challenged with NE.

## Data Availability Statement

The 16S rRNA gene amplicon sequencing results were submitted to the Sequence Read Archive of the NCBI (accession number: PRJNA766165). This data can be found here: https://www.ncbi.nlm.nih.gov/bioproject/766165.

## Ethics Statement

The animal study was reviewed and approved by China Agricultural University Animal Care and Use Committee (Beijing, China).

## Author Contributions

BS designed and performed experiments, analyzed data, and wrote the paper. PL and SY performed some experiments, and analyzed some data. YL and MG analyzed some 16SrRNA sequencing data. HL analyzed some metabolome data. ZL performed some experiments. YG initiated the study, designed animal experiments, analyzed data, and wrote the paper. All authors contributed to the article and approved the submitted version.

## Funding

This study was funded by the China Agriculture Research System program (CARS-41-G11).

## Conflict of Interest

Author HL is employed by Beijing Centre Biology Co., Ltd.

The remaining authors declare that the research was conducted in the absence of any commercial or financial relationships that could be construed as a potential conflict of interest.

## Publisher’s Note

All claims expressed in this article are solely those of the authors and do not necessarily represent those of their affiliated organizations, or those of the publisher, the editors and the reviewers. Any product that may be evaluated in this article, or claim that may be made by its manufacturer, is not guaranteed or endorsed by the publisher.

## Abbreviations

AhR, Aryl hydrocarbon receptor; APS, Astragalus polysaccharide; BWG, Body weight gain; Con A, Concanavalin A; DTT, 1,4-DL-Dithiothreitol; ELISA, Enzyme-linked immuno sorbent assay; FBS, Fetal bovine serum; FI, Feed intake; Foxp3, Forkhead transcription factor p 3; GAPDH, Glyceraldehyde-3-phosphate dehydrogenase; IFN-γ, Interferon-γ; IgA, Immunoglobulin A; IL-1β, Interleukin-1β; iNOS, inducible nitric oxide synthase; IRAK4, Interleukin-1 receptor associated kinase-4; LPL, Lamina propria lymphocytes; LPS, Lipopolysaccharide; LYZ, Lysozyme; MTT, A 3-(4,5-dimethylthiazol)-2,5-diphenyltetrazolium bromide; NE, Necrotic enteritis; NF-κB, Nuclear factor-κB; NLRP3, NOD-like receptor family proteins 3; NO, Nitrogen Oxide; PBMC, Peripheral blood mononuclear cell; PBS, Phosphate buffered saline; pIgR, Polymeric immunoglobulin receptor; PLS-DA, Partial least squares discrimination analysis; RT-PCR, Reverse transcription-rolymerase chain reaction; sIgA, Secretory immunoglobulin A; Tc cell, Cytotoxic T cell; TGF-β, Transforming growth factor-β; Th cell, Helper T cell; TLR2, Toll-like receptor; TNF-α, Tumor necrosis factor α; Treg cell, Regulatory cell; TSC, Tryptone-sulfite-cycloserine agar medium; V/C, Villi height/crypt depth; ZO-1, Zonula occludens-1.
